# Placeholder factors in ribosome biogenesis: please, pave my way

**DOI:** 10.15698/mic2017.05.572

**Published:** 2017-04-27

**Authors:** Francisco J. Espinar-Marchena, Reyes Babiano, Jesús Cruz

**Affiliations:** 1 Instituto de Biomedicina de Sevilla (IBiS), Hospital Universitario Virgen del Rocío/CSIC/Universidad de Sevilla, and Departamento de Genética, Universidad de Sevilla, E-41013, Seville, Spain.; 2 Present address: Swiss Institute for Experimental Cancer Research (ISREC), School of Life Sciences, Ecole Polytechnique Fédérale de Lausanne (EPFL), CH-1015 Lausanne, Switzerland.

**Keywords:** ribosome assembly, ribosomal proteins, RNA mimicry, trans-acting factors, yeast

## Abstract

The synthesis of cytoplasmic eukaryotic ribosomes is an extraordinarily energy-demanding cellular activity that occurs progressively from the nucleolus to the cytoplasm. In the nucleolus, precursor rRNAs associate with a myriad of *trans*-acting factors and some ribosomal proteins to form pre-ribosomal particles. These factors include snoRNPs, nucleases, ATPases, GTPases, RNA helicases, and a vast list of proteins with no predicted enzymatic activity. Their coordinate activity orchestrates in a spatiotemporal manner the modification and processing of precursor rRNAs, the rearrangement reactions required for the formation of productive RNA folding intermediates, the ordered assembly of the ribosomal proteins, and the export of pre-ribosomal particles to the cytoplasm; thus, providing speed, directionality and accuracy to the overall process of formation of translation-competent ribosomes. Here, we review a particular class of *trans*-acting factors known as "placeholders". Placeholder factors temporarily bind selected ribosomal sites until these have achieved a structural context that is appropriate for exchanging the placeholder with another site-specific binding factor. By this strategy, placeholders sterically prevent premature recruitment of subsequently binding factors, premature formation of structures, avoid possible folding traps, and act as molecular clocks that supervise the correct progression of pre-ribosomal particles into functional ribosomal subunits. We summarize the current understanding of those factors that delay the assembly of distinct ribosomal proteins or subsequently bind key sites in pre-ribosomal particles. We also discuss recurrent examples of RNA-protein and protein-protein mimicry between rRNAs and/or factors, which have clear functional implications for the ribosome biogenesis pathway.

## INTRODUCTION

Ribosomes are complex ribonucleoprotein organelles that are responsible for protein synthesis. In all organisms, ribosomes are composed of two ribosomal subunits (r-subunits), the large one (LSU) being about twice the size of the small one (SSU) 1. The production of ribosomes is an extraordinarily complicated cellular challenge. All organisms invest an important percentage of their resources to produce and subsequently assemble the individual constituents of the ribosomes, ribosomal RNAs (rRNAs) and ribosomal proteins (r-proteins), which must be represented in equimolecular amounts 2,3,4. This process, known as the ribosome biogenesis pathway, is a highly coordinated process that, in addition to the rRNAs and r-proteins, involves RNA and protein *trans*-acting factors. *Trans*-acting factors transiently bind to pre-ribosomal particles in a distinctive spatiotemporal manner and have precise functions during (i) the transcription, processing and chemical modification of the precursor rRNAs (pre-rRNAs); (ii) the folding and rearrangements of the pre-rRNAs within the pre-ribosomal particles; (iii) the synthesis, dedicated chaperoning, nuclear import (only in eukaryotes), assembly and repositioning within pre-ribosomal particles of the different r-proteins; (iv) also in eukaryotes, the intranuclear transport, acquisition of export competence and the exit of pre-ribosomal particles to the cytoplasm, as well as the cytoplasmic maturation steps that newly synthesized ribosomes must undergo before entering translation.

In bacteria, only about a dozen of protein *trans*-acting factors have been reported to participate in ribosome biogenesis 4,5,6. In archaea, about 50 small RNAs (i.e. modification guide small RNAs) and 40 protein *trans*-acting factors have been described 5,7,8. In eukaryotes, however, ribosome biogenesis has clearly increased its complexity. Thus, in the yeast *Saccharomyces cerevisiae*, about 80 small nucleolar RNAs (snoRNAs) and more than 250-300 protein *trans*-acting factors are currently known to participate in this process 9,10. In humans, about 300 snoRNAs and more than 600 protein factors have been shown so far to be required for the biogenesis of ribosomes 11,12.

Ribosome synthesis is clearly a directional process 13. Thus, most reactions occur irreversibly during the maturation of pre-ribosomal particles, among them, the pre-rRNA processing steps, the pre-rRNA folding, the snoRNA-dependent modifications, and the stepwise exchange of some protein *trans*-acting factors (for a general scheme of the ribosome biogenesis process, see Figure S1). Eukaryotes have evolved a nucleus, therefore, pre-ribosomal particles must travel across the nucle(ol)us and, after the acquisition of export competence, exit to the cytoplasm. Different factors are part of a quality control machinery that allows export-competent pre-ribosomal particles to associate with transport factors, which mediate their interactions with the nuclear pore complexes and their transport to the cytoplasm 14. Moreover, although it has been suggested that some translation might occur in the nucleus 15, it seems clear that, at least, nascent nuclear pre-ribosomal particles are incompetent for translation until the release of the last protein *trans*-acting factors in the cytoplasm and the assembly of the last r-proteins 16,17,18. These events, the displacement of the last factors and the assembly of the last r-proteins in the cytoplasm, are apparently prerequisites to confer translational competence to r-subunits (i.e. 19,20,21).

Several protein *trans*-acting factors have been reported that act as placeholders. The word "placeholder" has different meanings, among others it refers to someone who occupies a professional position on behalf of someone else. In Molecular Biology, a placeholder corresponds to a factor that temporarily binds a target until its replacement by a second factor, which binds to the same target normally with a higher affinity. Placeholder factors have been described to participate in different cellular processes, including chromatin remodelling and transcription (e.g. 22,23,24). In this review, we outline the current knowledge about placeholder factors involved in the biogenesis of ribosomes, focusing primarily on those from the yeast *S. cerevisiae*, in which this process has been most extensively studied. These factors include *trans*-acting factors, such as Mrt4 or Rlp24, which are paralogous placeholders of distinct r-proteins, or others *trans*-acting factors, such as Arx1, Nog1, Nog2/Nug2 or Tsr1, whose replacement pairs are not r-proteins. We also examine the role of distinct adaptors and chaperones, such as Rrb1, Sqt1 or Yar1, which recognize domains on their r-protein partners that are normally involved in binding to rRNAs. This latter phenomenon also resembles that known as RNA mimicry, by which some factors, such as Fap7 or Syo1, interact with a specific r-protein through the establishment of a protein interface that imitates part of the rRNA-binding surface of these r-proteins. We emphasise how all these factors, whose functions during ribosome biogenesis expand in many cases beyond their placeholding activity, and strategies render properly assembled r-subunits competent for translation.

## THE CLASSICAL VIEW: PARALOGUES OF RIBOSOMAL PROTEINS

In yeast, most r-proteins genes are duplicated and encode identical or nearly identical paralogous r-proteins, which, apparently in numerous cases, are functionally redundant 25. Strikingly, few r-proteins have additional paralogues that share extensive identity and similarity to them (e.g. 26,27,28). These paralogues, also known as ribosomal-like proteins, are not natural components of mature ribosomes and are unable to functionally replace their r-protein counterparts, even when overexpressed (e.g. 26,29), but interestingly, all of them have a role during ribosome biogenesis 28,30,31,32. This fact prompted S. J. Baserga to propose that each ribosomal-like protein could act as a placeholder for its paralogous r-protein on the pre-rRNA. A placeholder factor acts by preventing the premature assembly of its r-protein counterpart on its rRNA binding site, which, both proteins, considering their extensive homology, may share 31.

Ribosomal-like proteins include Imp3, Mrt4, Rlp7 and Rlp24, which display considerable sequence homology to r-proteins S9 (uS4 according to the recently proposed r-protein nomenclature 33), P0 (uL10), L7 (uL30) and L24 (eL24), respectively. In addition, part of the Nob1 endonuclease exhibits significant homology to S26 (eS26) 17 (Figure S2). Moreover, several *trans*-acting factors have gained ancient RNA-binding motifs in their structures that resemble those present in distinct r-proteins; for instance, Rrp5 contains 12 tandem S1 (bS1) RNA-binding motifs in its N-terminal domain 34; Snu13 is member of a family of K-turn binding proteins that also includes human r-proteins L7A (eL8) and S12 (eS12), and yeast r-protein L30 (eL30) 35. In this section, we discuss the functional relationship of Mrt4, Rlp24, Rlp7 and Imp3 with their paralogous r-proteins. The implications of the homology between Nob1, Rrp5 or Snu13 and their respective r-protein counterparts will not be discussed. In these three cases, it remains to be determined whether the *trans*-acting factors could have a placeholder activity.

### Mrt4 and Mex67 versus P0

**Figure 1:**
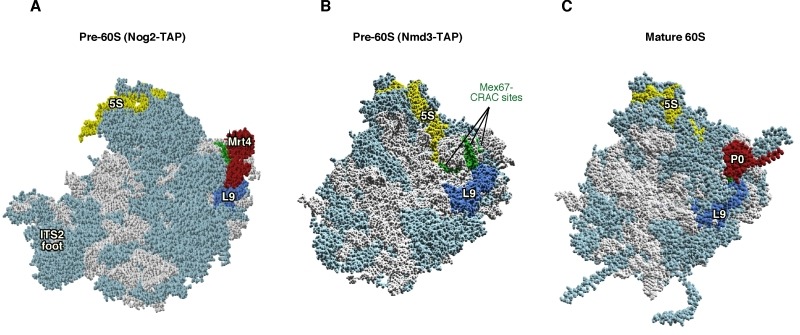
Figure 1: Mrt4 and Mex67 act as placeholder factors for the P0 r-protein. **(A)** Position of Mrt4 (red) in the early pre-60S r-particles purified with Nog2-TAP (PDB ID: 3JCT; 40). **(B)** Mex67-binding sites at the P0 neighbourhood (green), identified by CRAC 42, have been highlighted in the late/cytoplasmic Nmd3-TAP pre-60S r-particle (PDB ID: 5H4P; 43. **(C)** Position of P0 (red) in the mature 60S r-subunit (PDB ID: 3U5I, 3U5H; 44). Particles are viewed from the subunit interface slightly turned to the left. For orientation, the positions of the 5S rRNA (yellow), the L9 r-protein (royal blue) and the above CRAC sites of Mex67 (green) have been highlighted in the three structures. The ITS2 foot has also labelled in A. Note that some of the CRAC sites of Mex67 overlap with Mrt4 and P0 in A and C, respectively. The rest of rRNAs are coloured in pale blue and the rest of r-proteins and/or factors in light cornflower blue. Images were generated using the UCSF Chimera program (www.cgl.ucsf.edu/chimera).

From all these examples, perhaps the best-studied, homology-sharing pair of proteins consists of Mrt4 and P0. Mrt4 is homologous to the N-terminal domain of P0 (Figure S2), which corresponds to the rRNA binding domain of the r-protein 36. P0 has an additional C-terminal extension that is exposed to the solvent and interacts with the acidic P1 and P2 r-proteins and translation elongation factors 37. A few years ago, we could show that yeast P0 and Mrt4 are unable to bind simultaneously to r-particles by analysing the presence of either protein in complexes purified using functional TAP-tagged Mrt4 or P0 as affinity baits, respectively 29. This observation, together with the fact that a Mrt4-P0 chimera protein, containing as N-terminal domain the Mrt4 ORF, is able to partially complement the otherwise lethal absence of P0 29, and that a truncated P0 r-protein lacking its C-terminal domain functionally resembles Mrt4 38, strongly suggest that P0 and Mrt4 compete for the same rRNA site in r-particles, thus, successively occupying this site during LSU maturation 29. A similar scenario has been reported for human Mrt4 and P0 39. In full agreement with this hypothesis, the cryo-electron microscopy (cryo-EM) reconstruction of two distinct yeast pre-60S r-particles, which carry Mrt4 but lack P0, has revealed that indeed in these particles Mrt4 unequivocally localizes to a position equivalent to the one of P0 in the P-stalk of the mature LSU (Figures 1A and 1C) 40,41. Strikingly, theoretical estimation of the free RNA binding energy of both proteins suggests that P0 might bind to its rRNA site a little tighter than Mrt4 29, a fact whose biological significance will be further discussed.

The dynamics of the sequential exchange reaction of Mrt4 with P0 have been studied *in vivo*. Different evidence indicates that Mrt4 is a nucleo-cytoplasmic shuttling assembly factor, which associates with early to intermediate pre-60S r-particles and predominantly dissociates from late, cytoplasmic pre-60S r-particles 20,45. The replacement of Mrt4 by P0, thus, takes place mostly in the cytoplasm, although it could also occur in the nucleus 38. This replacement is a prerequisite to recycle Mrt4 back to the nucle(ol)us 20. How exactly this reaction takes place mechanistically is still unknown. Moreover, the exchange does apparently not occur directly, but instead, requires the participation of Yvh1, which is another nucleo-cytoplasmic shuttling assembly factor, non-homologous to either Mrt4 or P0 that co-enriches with late/cytoplasmic pre-60S r-particles 42,45,46. Interestingly, Yvh1-containing r-particles do neither contain Mrt4 nor P0 and equivalent results are obtained in reciprocal experiments 42,45,46. However, whether Yvh1 competes with the rRNA-binding site of Mrt4 and P0 has not been addressed until very recently 42. It has been shown that the stable association of Yvh1 with pre-60S r-particles depends on the r-protein L12 (uL11), which is the closest neighbour of P0 at the base of the P-stalk 44,47. However, still in the absence of L12, there is apparently no difference in the efficiency of Mrt4 re-importation to the nucleus (48, and our unpublished results). In conclusion, despite the fact that yeast Mrt4 and Yvh1 are non-essential proteins under standard laboratory conditions, both factors may play important roles controlling the position and timing of the assembly of P0, simultaneously providing a surveillance point to ensure that only mature LSUs can engage in translation (see below; further discussed in 14,17,20).

Interestingly, the Hurt laboratory has recently described that the nuclear-export factor Mex67 is another placeholder of the P0 r-protein 42, even though, Mex67 barely displays sequence homology with either Mrt4 or P0 (Figure S2). This group has identified that the heterodimeric Mex67•Mtr2 complex, which is involved in the export of late pre-60S r-particles 49,50, binds *in vitro* at two distant positions on Yvh1-purified pre-60S r-particles; the first one overlaps with the rRNA-binding site within the 5.8S rRNA of the RNA helicase Mtr4/Dob1, which is a cofactor of the exosome complex responsible of the 3' end maturation of 7S pre-rRNAs to 5.8S rRNAs (51; for a review, see 52); strikingly, the second position overlaps with the binding site of Mrt4 and P0 in pre-60S r-particles and the mature LSU, respectively (Figure 1B) 42. Remarkably, it could be shown that the Mex67•Mtr2 complex can hardly bind late pre-60S r-particles containing Mrt4 *in vitro*; this result is in agreement with a competition between Mex67•Mtr2 and Mrt4 for the same binding site if assuming only a minor contribution of the Mex67•Mtr2 rRNA-binding site at the 5.8S rRNA in these particles 42. Moreover, the structural characterization of Yvh1-containing pre-60S r-particles by cryo-EM reveals that Yvh1 binds adjacent to L12, a position that is close to but apparently not mutually exclusive to those of Mex67•Mtr2, Mrt4 or P0 42.

Taken together, the following model for the timing of P0 assembly has been proposed: (i) Mrt4 binds first at the incipient P-stalk site of nuclear pre-60S r-particles. (ii) Later, but still in the nucleus, the heterodimer Mex67•Mtr2 bound to Yvh1 replaces Mrt4 in the pre-60S r-particles; then, Mex67•Mtr2 acts as one of the RanGTP-independent factors involved in the export of pre-60S r-particles to the cytoplasm; (iii) once in the cytoplasm, the assembly of P0 occurs concomitantly to the release of Mex67•Mtr2 and Yvh1 from the base of the P-stalk in the cytoplasmic pre-60S r-particles. How can we reconcile this model with apparently contradictory findings indicating that the release of Mrt4 takes place mostly in the cytoplasm? The answer is not obvious, but the scenario clearly suggests the existence of alternative pathways to perform the same reaction. Moreover, as nuclear export of pre-60S r-particles is mediated by several redundant but cooperative systems (reviewed in 14) and Yvh1 is a non-essential factor 45,46, it is conceivable that Mrt4 may exchange in the cytoplasm, not only independently of Mex67•Mtr2, but also of Yvh1.

### Rlp24 versus L24

**Figure 2:**
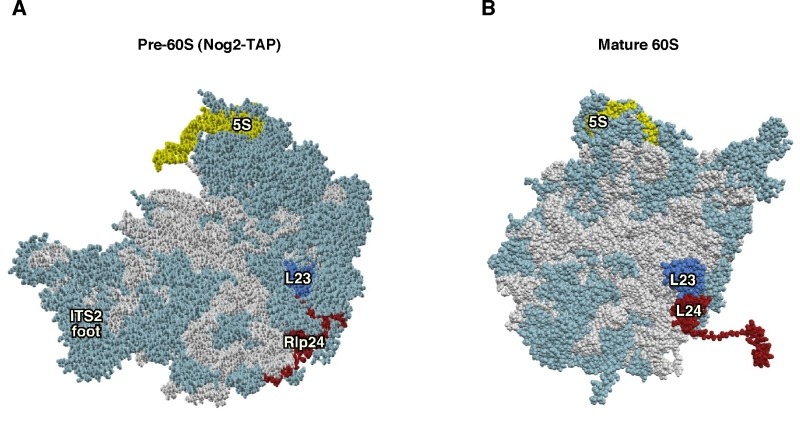
Figure 2: Rlp24 functions as a placeholder factor for the L24 r-protein. **(A)** Position of Rlp24 (red) in the early pre-60S r-particles purified with Nog2-TAP (PDB ID: 3JCT; 40). **(B)** Position of L24 r-protein (red) in the mature 60S r-subunit (PDB ID: 3U5I, 3U5H; 44). Particles are viewed from the subunit interface. For orientation, the positions of the 5S rRNA (yellow) and the L23 r-protein (royal blue) have been highlighted. The ITS2 foot has also labelled in A. Note that the last ca. 50 amino acids from the C-terminal part of Rlp24 and ca. 20 amino acids from the C-terminal end of L24 could not be modelled in the respective structures. The rest of rRNAs are coloured in pale blue and the rest of r-proteins and/or factors in light cornflower blue.

Another well-studied paralogous pair comprised of a ribosomal-like protein and an r-protein is represented by the conserved eukaryotic *trans*-acting factor Rlp24 and the LSU r-protein L24. Rlp24 and L24 share the N-terminal domain, which for L24 corresponds to the region that binds to mature LSUs (Figure S2). Yeast Rlp24 belongs to the category of *trans*-acting factors known as B factors, which are required for the proper maturation of 27SB pre-rRNAs within intermediate pre-60S r-particles 53. L24 is a non-essential LSU r-protein, whose role in LSU biogenesis has so far not been properly characterized 28. In any case, there is enough evidence to conclude that Rlp24 functions as a bona fide placeholder for L24. First, L24 is not present in pre-60S r-complexes purified using TAP-tagged Rlp24 as a bait 28. Second, both proteins seem to recognize the same binding site on r-particles, as shown by cryo-EM studies of distinct nuclear pre-60S r-particles, which reveal that the density found at the location of L24 in these particles clearly corresponds to the N-terminal part of Rlp24 (Figure 2) 40,41. As a corollary of this, release of Rlp24 from pre-60S r-particles is a pre-requisite for assembly of L24.

The replacement of Rlp24 with L24 occurs in the cytoplasm following different steps: (i) Rlp24, most likely assisted by the WD-40 repeat protein Mak11, associates in the nucleolus with very early pre-60S r-particles 28,54. This reaction appears to be coupled to the recruitment of the GTPase Nog1 to pre-60S r-particles, which directly and specifically interacts with Rlp24 28,40. (ii) Upon arrival in the cytoplasm, the AAA-ATPase Drg1, which forms hexamers in the presence of ATP, binds to the exported pre-60S r-particles and allows the specific dissociation of Rlp24. This dissociation step is a prerequisite for the subsequent cytoplasmic maturation steps of these pre-60S r-particles, including the release of other shuttling factors such as Nog1 and Bud20 and the recruitment of later-acting cytoplasmic factors such as Rei1 55,56,57,58. A monomer of Drg1 is composed of an N-terminal domain followed by two consecutive AAA-ATPase domains: D1 and D2 (reviewed in 59). *In vitro*, Drg1 binds specifically and directly to the C-terminal domain of Rlp24, which is not conserved in L24 60; consistently, expression of a truncated version of Rlp24 lacking the last 53 amino acids (Rlp24∆C) prevents the recruitment of Drg1 to pre-60S r-particles *in vivo*, and, as a consequence, Rlp24∆C is not properly released from cytoplasmic pre-60S r-particles and final maturation of nascent LSUs cannot be completed 55. *In vitro*, the C-terminal domain of Rlp24 also stimulates ATP hydrolysis in both AAA domains of Drg1 60; while ATP hydrolysis in the D2 domain triggers the dissociation of Rlp24 from pre-60S r-particles, ATP hydrolysis in the D1 domain is required for the subsequent release of Drg1 from Rlp24 and likely the dissociation of the Drg1 hexamer into monomers 60,61. Interestingly, it has been shown that Drg1 also directly binds the FG-repeat nucleoporin Nup116, and more importantly, that this interaction optimizes the release of Rlp24 from pre-60S r-particles, suggesting some coupling between the export of pre-60S r-particles and the initiation of their cytoplasmic maturation 60. (iii) Finally, Rlp24 is recycled back to the nucle(ol)us and L24 stably assembles into pre-60S r-particles. However, in clear contrast to P0, which is required for the efficient release of its placeholder Mrt4 from pre-60S r-particles 20, likely indirectly by its role in dissociating Yvh1 and the Mex67•Mtr2 complex from those particles 55, it appears that L24 does not contribute to the release of Rlp24 from pre-60S r-particles. Thus, the complete absence of L24, by the double deletion of the *RPL24A* and *RPL24B* genes, does not lead to a failure in either the release or the subsequent nucleolar recycling of Rlp24 (cited as unpublished results in 28).

The cytoplasmic assembly of L24 appears to be coupled to the recruitment of the non-essential factor Rei1 to pre-60S r-particles 62. Rei1 is highly homologous to Reh1, and, it has been shown that both factors have a partially redundant function during maturation of nascent LSUs 62,63. Rei1 directly interacts with the J-domain protein Jjj1, which recruits and activates the Hsp70-type ATPase Ssa1-Ssa2 64,65,66. Different authors have shown that one of the primary roles of Rei1 is the release and nuclear recycling of the heterodimeric Arx1•Alb1 complex from cytoplasmic pre-60S r-particles, although it is not known how this reaction mechanistically occurs 64,66,67. Moreover, this activity has indeed been questioned and attributed to either Jjj1 and Ssa 68 or Reh1 43, which both have been suggested to release simultaneously Rei1 and Arx1. The recent cryo-EM characterization of r-particles containing Rei1, Arx1 and Jjj1 or with Rei1, Arx1 and Alb1 at near-atomic resolution 68,69 will allow the development of models that clearly will help to solve this question. These and others models derived from cryo-EM reconstruction analyses of selected pre-60S r-particles (e.g. see 70), unambiguously show that Arx1 binds near the solvent-exposed side of the polypeptide exit tunnel (PET), suggesting that it could function as a placeholder for different nascent chain-associated factors, including methionine aminopeptidases (MetAPs) (discussed later). Interestingly, these analyses also reveal the global structure of Rei1 on r-particles, notably showing that its C-terminal segment penetrates into the PET and extends almost up to the peptidyl transferase centre (PTC) 68. Strikingly, this segment is structurally homologous to the C-terminal extension of the GTPase Nog1 40 and to the one of Reh1 43, which could also similarly insert into the PET; thus, the binding of these three factors to pre-60S r-particles is mutually exclusive and, as it will be discussed later, confers directionality to the cytoplasmic LSU maturation. These findings therefore indicate that Nog1 is a placeholder factor for Rei1, and in turn, Rei1 a placeholder factor for Reh1. Alternatively, Rei1 and Reh1 may have redundant functions and Nog1 could function as placeholder for either factor (see below).

## THE EXCEPTIONS

Not all ribosomal-like proteins act as placeholders of their respective paralogous r-proteins. Indeed, different studies on the *trans*-acting factors Rlp7 and Imp3 clearly contradict the intuitive hypothesis that these factors could compete with their counterpart r-proteins L7 and S9, respectively, for the same binding sites on the pre- or mature rRNAs.

### Rlp7 versus L7

**Figure 3:**
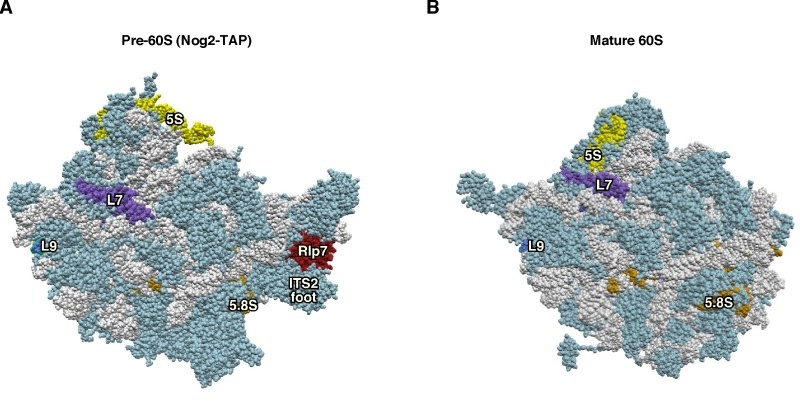
Figure 3: Rlp7 is not the placeholder factor for the assembly of L7 r-protein. **(A)** Position of Rlp7 (red) and L7 (purple) in the early pre-60S r-particles purified with Nog2-TAP (PDB ID: 3JCT; 40). **(B)** Position of L7 r-protein (purple) in the mature 60S r-subunit (PDB ID: 3U5I, 3U5H; 44). Particles are viewed from the solvent side. For orientation, the positions of the 5S rRNA (yellow), 5.8S rRNA (gold) and the L9 r-protein (royal blue) have been highlighted. The ITS2 foot has also labelled in A. Note that L7 is found at its final assembly position within Nog2-TAP pre-60S r-particles. The first ca. 20 amino acids of both proteins could not be modelled in either structure. The rest of rRNAs are coloured in pale blue and the rest of r-proteins and/or factors in light cornflower blue.

Our group, in collaboration with that of M. Fromont-Racine and A. Jacquier, reported a few years ago that Rlp7 and L7 could coexist in the same pre-60S r-particles; consistently, cross-linking and cDNA analysis (CRAC) experiments demonstrated that the Rlp7 and L7 binding sites are actually distinct in pre- and/or mature rRNAs and distant enough from each other that they do not result in steric binding interference 71. Similar findings were independently obtained by the laboratory of J. L. Woolford, Jr. 72. Rlp7, which is 78 amino acids longer than L7, shares a considerable overall sequence and structure homology with L7, except in its N-terminal region, its internal loop and few other discrete regions (Figure S2). Rlp7 belongs to the group of proteins known as A3 assembly factors 10, which hierarchically and interdependently associate with early pre-60S r-particles and are globally required for optimal 5' to 3' exonucleolytic trimming of the 27SA3 pre-rRNA to the 27SBS pre-rRNA, a processing step that generates the 5' end of mature 5.8SS rRNA 10,31,52,73,74. However, the rRNA-binding site of Rlp7, as those of the other A3 assembly factors, maps to positions in the ITS2 spacer instead of positions close to the 5' end of 27SA3 pre-rRNA in the ITS1 spacer 71,2,75. It has been suggested that the A3 assembly factors may play structural roles in chaperoning ITS2 within pre-60S r-particles, thus protecting 27S pre-rRNAs from rapid turnover and facilitating their correct processing (further discussed in 10,73,75, see also 51). Cryo-EM analyses have confirmed the rRNA-binding sites of Rlp7 and several other A3 factors. These sites cluster around ITS2, providing an explanation to the interdependent association of A3 factors with pre-60S r-particles 40,41. However, in the crystal structure of mature LSUs, the globular domain of L7 binds domain II of 25S rRNA, as well as 5S rRNA, while its N-terminal extension interacts with the expansion segment ES7 of 25S rRNA (Figures 3A and 3B) 44,47. In agreement with our data 71, the L7 interactions already exist in pre-60S r-particles, as also revealed by cryo-EM analyses of selected pre-60S particles (Figures 3A and 3B) 40,41. Curiously, it has been shown that L7 is also required for 27SA3 pre-rRNA processing 76,77,78. It seems that the assembly of L7, as that of any of its neighbouring r-proteins in rRNA domain II, such as L4 (uL4), L6 (eL6), L14 (eL14), L16 (uL13), L18 (eL18), L20 (eL20), L32 (eL32) and L33 (eL33), allows the stabilisation of rRNA structures within pre-60S r-particles, which is a prerequisite for the stable association and function of A3 assembly factors (77,79, further discussed in 80).

Likely, Rlp7 has evolved from the highly conserved L7 r-protein to use a similar recognition motif to bind and function at a different location during LSU maturation. However, it is unclear how proteins showing apparently very similar architectures could be specifically targeted to different places in pre-ribosomal complexes, instead of competing for the same RNA substrates. As a possibility, we can imagine a scenario in which distinct co-factors specifically recruit the *trans*-acting factor or the corresponding paralogous r-protein to the pre-rRNAs at their different RNA binding sites and help their stable association or assembly.

### Imp3 versus S9

**Figure 4:**
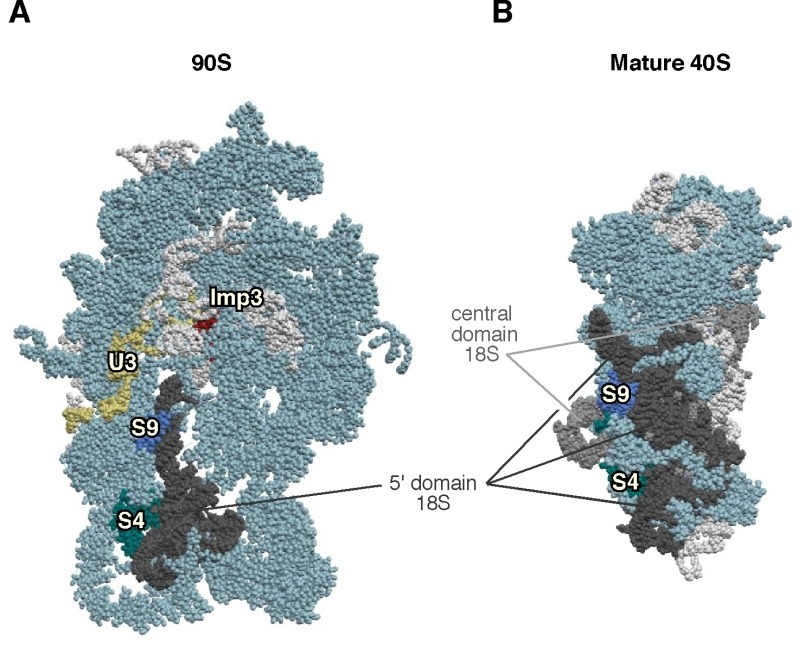
Figure 4: Imp3 is not the placeholder factor for the assembly of S9 r-protein. **(A) **Position of Imp3 (red) and S9 (medium blue) in the 90S pre-ribosomal particle, also known as the SSU processome (PDB ID: 5TZS; 93). **(B) **Position of S9 r-protein (royal blue) in the mature 40S r-subunit (PDB ID: 3U5B, 3U5C; 46). Mature 40S r-subunit is seen from the A-site view, and the 90S pre-ribosomal particles has been consequently oriented from a similar position regarding the nascent 40S r-subunit. The positions of the U3 snoRNP (yellow) and that of the S4 r-protein (cyan) have been highlighted. The 5' domain of 18S rRNA has been coloured in dark grey, the central domain in medium grey, and the rest of 18S rRNAs in pale blue; r-proteins and/or factors have been coloured in light cornflower blue.

Imp3 is a paralogue of the SSU r-protein S9. The similarity of these two proteins extends all over their complete sequences (Figure S2), including their putative, conserved RNA-binding domains 32. The functional role of Imp3 has been investigated *in vitro* and *in vivo*; Imp3 is an essential *trans*-acting factor required for SSU biogenesis, more specifically for the cleavage at the early sites A0, A1 and A2 in the 35S pre-rRNA within 90S pre-ribosomal particles 32. It has also been shown that Imp3, together with the factors Mpp10 and Imp4, forms a stable sub-complex 32,81, which co-transcriptionally associates with the 5'-ETS region of 35S pre-rRNA 82,83,84. *In vitro* experiments have suggested that the association of this sub-complex with 90S pre-ribosomal particles is required to mediate and stabilize specific base-pair interactions of residues in 5' ETS with the hinge region on the 5' end of the U3 snoRNA 85,86; these base pairings are indeed critical for *in vivo* ribosome maturation (reviewed in 87). In turn, S9 is an essential r-protein, which is also required for early pre-rRNA processing at the A0-A2 sites 88. In yeast and humans, S9 is a primary binding r-protein that assembles likely co-transcriptionally to the body of the SSU 89,90, more specifically at positions corresponding to helices H3, H12, H17, and expansion segment ES6S of the 18S rRNA, while also interacting with other SSU r-proteins such as S2 (uS5), S4 (eS4), S24 (eS24) and S30 (eS30) (Figure 4) 44,91. The cryo-EM reconstruction of 90S pre-ribosomal particles from both *S. cerevisiae* and *Chaetomium thermophilum*, an ascomycete related to *S. cerevisiae* 92, has recently been obtained 93,94,95. These reconstructions have allowed the identification of the position of many *trans*-acting factors from these particles, among them, Imp3 and about a dozen r-proteins from the SSU. Some of these r-proteins have consistently been identified as stable components of purified 90S pre-ribosomal particles 96,97,98. In the reconstructions, Imp3 binds to Mpp10 and Imp4, and this latter approaches the 5' part of U3 snoRNP. In agreement with its early assembly, S9 was found among those SSU r-proteins identified in the 90S r-particles. Importantly, S9 appears to be bound to its final rRNA-binding site within the nascent 18S rRNA, adopting a mature-like conformation in the particles (Figure 4) 93,94. In conclusion, all these data indicate that Imp3, despite its similarity to S9, is not its placeholder factor in 90S pre-ribosomal particles.

## NEW PLACEHOLDER FACTORS: THE MOST RECENT DATA

In the last ten years, the use of CRAC and cryo-EM methodologies has permitted gaining information concerning the rRNA-binding sites and the location of a considerable number of *trans-*acting factors, especially from yeast, within pre-ribosomal particles. These achievements, together with the information available on the structure and location of all r-proteins revealed by crystal structures of ribosomes or r-subunits of different eukaryotes, the compositional analysis of pre-ribosomal particles and, importantly, the extended body of genetic and biochemical data on the role of *trans*-acting factors and r-proteins, are providing clues into the mechanistic details of the ribosome assembly process for the first time at high resolution. Particularly relevant to the scope of this review has been the discovery of many other examples of placeholder factors that mask particularly important ribosomal sites until a specific r-subunit maturation event has been accomplished. In this section, we enumerate several examples of placeholder factors that we consider to have a clear biological relevance, focusing on those that block the recruitment of essential translation factors or r-proteins to cytoplasmic pre-ribosomal intermediates, thus, ensuring that only mature r-subunits engage in protein synthesis.

### Tsr1 blocks binding of both the GTPase eIF5B and the ATPase Rio1 to late pre-40S ribosomal particles

Tsr1 is an essential conserved *trans*-acting factor required for efficient nucleo-cytoplasmic transport of pre-40S r-particles and processing of 20S pre-rRNA to mature 18S rRNA 99,100. Tsr1 has been shown to be recruited to early pre-40S r-particles in the nucleolus and accompany them together with a few other *trans*-acting factors (Dim1, Enp1, Ltv1, Pno1/Dim2, Nob1, and Rio2) to the cytoplasm. There, Tsr1 rapidly dissociates and is recycled back to the nucleolus 100,101. Interestingly, Tsr1 is structurally related to translational GTPases such as EF-Tu or eIF5B/Fun12 in their GTP-bound form. However, Tsr1 neither is a GTP-binding protein nor has GTPase activity 102. As expected from this structural similarity, cryo-EM and CRAC analyses confirmed that Tsr1 binds, albeit differently than standard translational GTPases 102, the GTPase centre site on pre-40S r-particles 21,102,103. In any case, the binding of Tsr1 to pre-40S particles is mutually exclusive with, at least, that of the GTPase eIF5B and the ATPases Rli1 and Rio1, which are all required for proper maturation of pre-40S r-particles to SSUs 21,102,104. Moreover, the position of Tsr1 on pre-40S r-particles potentially impedes joining of these particles to LSUs and occludes part of the mRNA channel 21,102.

In conclusion, the presence of Tsr1 on pre-40S r-particles is incompatible not only with maturation of SSU but also with translation. Thus, Tsr1 is a good example of a placeholder factor for a set of distinct factors that times key steps during SSU formation and function. How and when the dissociation of Tsr1 from pre-40S r-particles is triggered is still unknown.

It is worth mentioning that Tsr1 shares substantial sequence identity with another GTPase, Bms1 99. As Tsr1, Bms1 is an essential *trans*-acting factor involved in SSU biogenesis, but in contrast to Tsr1, Bms1 is required for pre-rRNA processing at the early sites A0, A1 and A2 99,105. Bms1 is a stable component of 90S pre-ribosomal particles 96 that likely binds co-transcriptionally to the nascent pre-rRNA 83,84,106 and apparently efficiently dissociates following the formation of early nuclear pre-40S r-particles 100. As mentioned above, Tsr1 seems to be recruited to these type of pre-40S r-particles. Interestingly, Bms1 has been unambiguously modeled into the cryo-EM structure of 90S pre-ribosomal particles 93,94,95. In one of these articles, the authors have claimed that the binding site of Bms1 overlaps significantly with that of Tsr1 and suggested that Bms1 could likely work as placeholder for Tsr1 during the transition of 90S to pre-40S ribosomal particles 95. However, whether or not release of Bms1 is linked to recruitment of Tsr1 is still unexplored.

### Molecular events involving Nmd3

Nmd3 is an essential conserved factor that connects pre-60S r-particles to the Crm1/Xpo1 exportin by its nuclear export sequence (NES), thereby enabling the RanGTP-dependent export of late pre-60S r-particles from the nucleus 107,108. Nmd3 exits to the cytoplasm associated with pre-60S r-particles where it is released and then recycled back 107. It has been demonstrated that a truncated version of the Nmd3 protein lacking its last 100 amino acids, which includes its NES but not its nuclear localization sequence (NLS), is able to bind pre-60S r-particles that remain trapped in the nucle(ol)us 107. Dissociation of Nmd3 from cytoplasmic pre-60S r-particles is imperative for r-subunit joining and translation initiation 109. Moreover, this reaction involves the activity of the cytoplasmic GTPase Lsg1/Kre35 and the assembly of L10 (uL16), which seems to stably lock into its final position on cytoplasmic pre-60S r-particles concomitantly to the removal of Nmd3 110,111. In line with this model, distinct mutations in *LSG1* or *RPL10* or depletion of L10 cause a retention of Nmd3 on cytoplasmic pre-60S r-particles and are synthetically lethal with specific *nmd3* mutants 111, while overexpression of wild-type Nmd3 or the presence of mutated Nmd3 versions with reduced affinity for pre-60S r-particles suppress the growth defect and the failure to recycle Nmd3 in *lsg1* and/or *rpl10* mutants 111,112.

**Figure 5:**
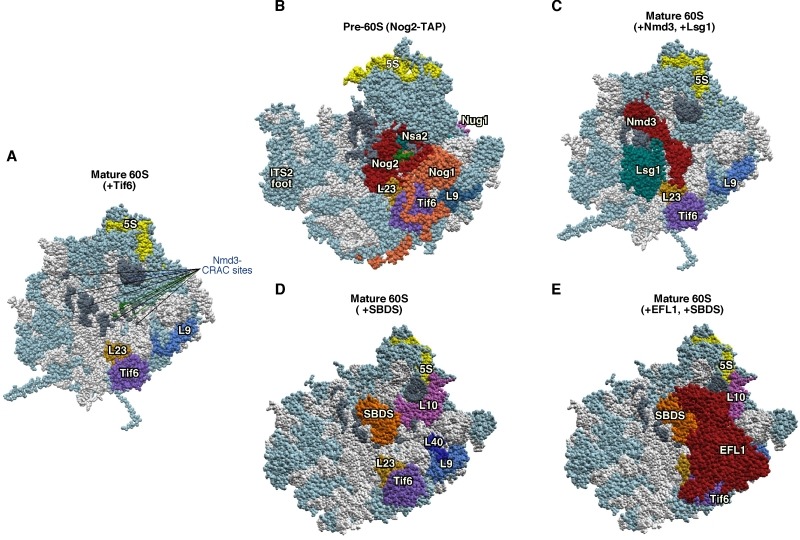
Figure 5: Interactions at the interface side of cytoplasmic pre-60S ribosomal particles. **(A) **The Nmd3-binding sites, identified by CRAC at 25S rRNA helices H38, H69-69 and H89-90 115, have been highlighted in dark grey in a reconstituted 60S r-subunit (PDB ID:5T62; 114). The CRAC sites common for Nmd3, Dbp10 and Nug1 115,118 are labelled in green. **(B) **Position of Nog2 (red), Nsa2 (blue), Nog1 (gold) and Nug1 (pink) in the early pre-60S r-particle purified with Nog2-TAP (PDB ID: 3JCT; 40). Note that only a very small portion of Nug1 has been resolved in this particle. **(C) **Position of Nmd3 and Lsg1 in a reconstituted 60S r-subunit (PDB ID:5T62; 114). Note that only the region of Nmd3 comprised between residues 46 and 388 of 518 in total is shown. The N-terminal end of Nmd3 approaches to Tif6 while the C-terminal end contact L1. In A-C, the position of Tif6 (purple) is shown. The locations of the 5S rRNA (yellow) and that of the L23 (gold) and L9 r-proteins (royal blue) have also been highlighted. **(D) **Position of SBDS (dark gold) in the 60S r-subunit. The structure of a pre-60S r-particle from *Dyctiostelium discoideum* containing Tif6 and reconstituted with human SBDS and EFL1 (PDB ID: 5ANB; 116) was superimposed on the structure of yeast 60S r-subunit (PDB ID: 5APN; 68); then, all common proteins from *D. discoideium* were removed from the model. L9 (royal blue), L40 (navy blue), L10 (violet), L23 (gold) and 5S rRNA (yellow) were highlighted. Note that SBDS is shown in its open conformation state. **(E) **As D, but additionally showing EFL1 (red) on top of Tif6. In all figures, the Nmd3-CRAC sites shown in A (dark grey) were also highlighted, the rest of rRNAs are coloured in pale blue and the rest of r-proteins and/or factors in light cornflower blue.

Cryo-EM analyses of purified mature LSUs harbouring *in vitro*-reconstituted MBP-tagged Nmd3 or of native Nmd3-purified pre-60S r-particles have shown that Nmd3 binds to the intersubunit face of pre-60S r-particles spanning from the L1 (uL1) stalk to the position where Tif6 binds (see later), going through the E- and P-sites (Figure 5C) and contacting helices H38 (also known as the A-site finger), H65 and H95, in addition to the sarcin/ricin loop (SRL) in 25S rRNA 43,113,114. These findings are consistent with the Nmd3-binding sites detected by the CRAC method (Figure 5A) 115. The association of Nmd3 to pre-60S r-particles seems to be very dynamic and, in unison with the L1-stalk, it could adopt several states 114. Most, if not all, of these states are incompatible with the simultaneous presence of the Sdo1•Efl1 complex in the r-particles (also discussed later) 114,116 or r-protein L40 (eL40), which, as L10, also assembles in the cytoplasm 117. In agreement, purified pre-60S r-particles purified via TAP-tagged Nmd3 clearly lack some r-proteins, among them L10, L40, L12 (uL11) and L41 (eL41) 43. Thus, due to the fact that mature LSUs contain r-proteins L10 and L40, it is expected that the position of Nmd3 in the *in vitro* reconstituted LSUs may not fully correspond with the one it adopt within native pre-60S r-particles 43,114 (see also Figure 5C). Moreover, as a corollary of the steric clash between Nmd3 and Sdo1•Efl1, it could be deduced that Lsg1 might not bind to the GTPase-associated centre (GAC) on pre-60S r-particles; indeed, the Hurt laboratory has suggested that Lsg1 could contact pre-60S r-particles also at the interface face of LSU but close to helix H69 of 25S rRNA and the P-site 42. These suggestions have been later confirmed by cryo-EM analysis of native or reconstituted Nmd3-containing particles 43,114.

These cryo-EM studies fully explain why r-subunit joining and translation are not possible as long as Nmd3 is not released from pre-60S r-particles. Moreover, they could also reveal that the C-terminal domain of Nmd3 adopts a structure that mimics that of translation elongation factor eIF5A and binds similarly as eIF5A to the E-site of pre-60S r-subunits 114. Importantly, these studies also revealed that most of the Nmd3-binding sites overlap with those of the GTPase Nog2 115, which have been determined by cryo-EM 40. In agreement, purification of pre-60S r-particles indicates that Nog2 and Nmd3 are not simultaneously present on the same pre-60S r-particles (Figure 5). Different experimental approaches indicate that the binding of Nog2 to early pre-60S r-particles precedes that of Nmd3 (e.g. 40), and consistently, the depletion of Nog2 causes the premature binding of Nmd3 to these particles 115.

Altogether, these experiments indicate that Nog2 is the placeholder factor of Nmd3, which acts by blocking the premature recruitment of the latter and, therefore, providing the time frame necessary for pre-60S r-particles to acquire their export competence. Interestingly, this activity seems to be coupled to that of other *trans*-acting factors, such as the AAA-ATPase Rea1 and its substrate Rsa4, in a way where stable binding of Rea1 and Rsa4 to pre-60S r-particles requires Nog2, and the release of Nog2 from pre-60S r-particles requires not only its GTPase activity, but also the ATPase activity of Rea1 115. Nmd3, on the other hand, supervises structurally and functionally the flexibility of the L1-stalk and the correct conformation of the E- and P-sites of LSUs; together with other ribosome assembly factors, such as Tif6, Nmd3 also impedes premature association of pre-60S r-particles with SSUs.

### Efl1•Sdo1 probes critical functional sites on pre-60S r-particles during LSU maturation

Efl1/Ria1 and Sdo1 (yeast SBDS orthologue) are quasi-essential *trans*-acting factors required for cytoplasmic LSU maturation 19,119,120. Efl1 is a cytoplasmic GTPase composed of five structural domains; it is highly homologous to the translation elongation eEF2 factor, which is responsible for the translocation reaction of the ribosome following each round of polypeptide elongation 1,19,119. In turn, Sdo1 is a very flexible protein, formed by three domains, which resembles bacterial ribosome recycling factor RRF 116,121. Both proteins are functionally related to each other, Tif6 and r-protein L10 19,119,121,122. Based on the structural similarity, it has been suggested that Efl1 interacts with pre-60S r-particles in a very similar manner as eEF2 does with 80S ribosomes; recruitment of Efl1 needs the presence of P0, the largest component of the ribosomal P0/P1/P2 stalk (uL10/P1/P2) 55 and the P-stalk base, the r-protein L12 (uL11), on the pre-60S r-particles 48; then, Sdo1-stimulated GTP hydrolysis might trigger a sort of translocation reaction that facilitates the dissociation and recycling of Tif6 (see 116,120,121,122, and references therein).

Recently, cryo-EM analyses of reconstituted pre-60S r-particles containing or lacking endogenous Tif6 from *Dictyostelium discoideum* and harbouring both human EFL1 and SBDS have allowed building models that deduce the precise interaction of these three factors with pre-60S r-particles (Figure 5). These models also provide a molecular scenario to understand how the release of Tif6 from these particles occurs 116. In this possible scenario: (i) first, SBDS is recruited to Tif6-containing late cytoplasmic pre-60S r-particles, in which the assembly of L10 and the P-stalk have already occurred. Tif6 is at its canonical position on the LSU interface bound to the C-terminal part of L23 (uL14) in the proximity of the SRL and the N-terminus of L24, thereby inhibiting r-subunit joining and, thus, preventing pre-60S r-particles from prematurely engaging in translation 47,123; the three domains of SBDS adopt a "closed conformation": domain I occupies the P-site of LSU and is in contact with a flexible loop of L10 (named P-site loop) 122, components of the PTC and the entrance of the PET; domain II mediates binding of SBDS to 25S rRNA, and domain III, which structurally resembles domain V of Efl1, contacts the SRL and the neighbouring base of the P-stalk 116. (ii) Second, EFL1 in its GTP-bound form binds the nascent GAC from the pre-60S r-particles, while contacting both Tif6 and SBDS, and r-proteins P0 and L12. Interestingly, the binding of domain III of SBDS on pre-60S r-particles is mutually exclusive with that of domain V of EFL1, thus, it seems that, upon EFL1 recruitment, SBDS undergoes a switch to reposition itself in a "open conformation" where its domain II rotates 60° relative to its domain I and, especially, its domain III rotates 180° away from the base of the P-stalk 116. (iii) These changes are supposed to accommodate EFL1 into the GAC leading to slight conformational adjustments that result in a more extensive binding of domain I of EFL1 to the SRL in the pre-60S r-particles; this new SRL-bound conformation overlaps with part of the Tif6-binding site and it is supposed to facilitate the release of Tif6 from these particles. (iv) The accommodated state of EFL1 also seems to stimulate the GTP hydrolysis of the factor, which causes another conformational change of EFL1 that destabilizes its interaction with pre-60S r-particles and that of SBDS; as a consequence, both SBDS and GDP-bound EFL1 dissociate from these Tif6-lacking pre-60S r-particles. Still, the exact role of GTP hydrolysis for Tif6 eviction remains to be elucidated 116.

Together, these findings indicate that Efl1 and Sdo1, as other above-mentioned examples, are factors with decisive roles in timing LSU maturation. Sdo1 examines the structure and function of important sites of LSUs, such as the PTC, the P-site and the entrance of the PET. Sdo1 seems to verify the assembly of L10. In turn, Efl1 proofreads the integrity of the GAC in LSUs, once the P-stalk has been properly assembled. Any delay or defect in the evaluation of all these sites would have as a consequence the inefficient release of Tif6 from pre-60S r-particles, thus, preventing translation by these apparently aberrant particles. Warren and co-workers assume that the Efl1•Sdo1-dependent maturation reaction might occur downstream of the assembly of L10 and the release of Nmd3 (107,108,111; for a review, see 17). This scenario differs from the one previously and currently reported by the Johnson laboratory, claiming that the release of Tif6 occurs prior to 18,55 or could be coupled with the Lsg1-mediated release of Nmd3 114, respectively. Future experiments are clearly required to clarify these issues.

### Nog1, Rei1 and Reh1 are plugs that block the nascent polypeptide exit tunnel of LSUs

**Figure 6:**
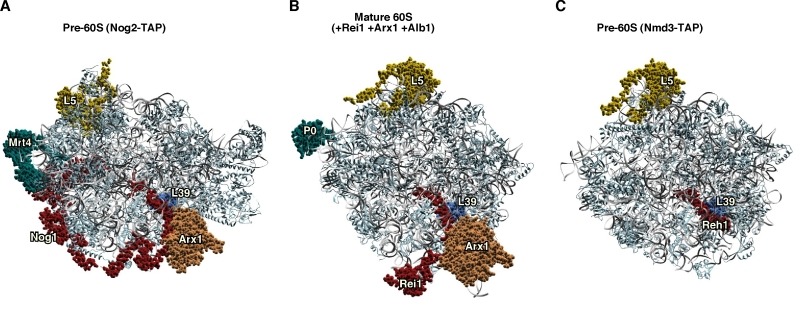
Figure 6: Interactions at the polypeptide exit tunnel of pre-60S ribosomal particles. **(A) **Position of Nog1 (red) and Arx1 (dark gold) in the early pre-60S r-particle purified with Nog2-TAP (PDB ID: 3JCT; 40). Note that the C-terminal part of Nog1 enters the tunnel. The location of Mrt4 (cyan) is also shown. **(B) **Position of Rei1 (red) and Arx1 (dark gold) in a reconstituted 60S r-subunit (PDB ID: 5APN; 68). Note that only the middle part (141-261) and the C-terminal end (300-393) of the protein is visualised. As above, the C-terminal part of Rei1 enters the tunnel. The location of P0 (cyan) is also shown. **(C) **Position of Reh1 (red) in the late/cytoplasmic pre-60S r-particles purified with Nmd3-TAP (PDB ID: 5H4P; 43). Note that only the region of the protein occluding the tunnel could be visualised (amino acids 377-432 of 432). Particles are viewed from the solvent side. For orientation, the locations of L5 (yellow) and L39 (blue) have been highlighted. The rest of rRNAs are coloured in pale blue and the rest of r-proteins and/or factors in light cornflower blue.

As previously mentioned, cryo-EM has allowed to deduce that Nog1 is the placeholder of Rei1 (for a review, see 124). Notably, both Nog1 and Rei1 enter the tunnel from the exit side with their C-terminal ends oriented to the PTC 40,68 (Figure 6A and B). This N- to C-terminal orientation resembles that of the nascent polypeptide chain during translation. Nog1 is replaced by Rei1 in the cytoplasm, however, the precise mechanism of this exchange is still elusive 55,56,57,58. Another protein, SBDS (Sdo1), is able to bind the exit tunnel, but in this case, the interaction takes place only via a very short extension, involving tunnel insertion of the N-terminal end of Sdo1, nearby the PTC 116. In a recent review, B. J. Greber has nicely modelled how SBDS and Rei1 interact without overlapping in the tunnel in an arrangement where the N-terminal end of SBDS is positioned close to the C-terminal end of Rei1, a circumstance that could not occur in the case of SBDS and Nog1 124. In this manner, the entire tunnel is filled, thus, providing a situation that could allow the complete functional proofreading of the integrity of this ribosomal site in cytoplasmic pre-60S r-particles.

Recently, the C-terminal helix of Reh1, which is highly homologous to that of Rei1 both at the level of sequence and structure, has also been found inserted in the PET of cytoplasmic pre-60S r-particles. The orientation of the C-terminal tail of Reh1 inside the tunnel is similar to that of the C-terminal extension of Rei1 (Figure 6C) 43. It seems that Reh1 binds downstream of Rei1 during the maturation of these particles, as suggested by the fact that Reh1-containing pre-60S r-particles lacks the Arx1•Alb1 complex 63.

### Nug1 and Dbp10: more overlapping interactions

The conformation of the nascent PTC is also verified by different factors during LSU maturation, which are expected to act sequentially on this site (Figure 5). First, distinct residues of the PTC are known to be subjected to pseudouridylation and 2'-O-methylation by specific snoRNPs (for an example, see 125), or base methylation by the site-specific methylases Spb1 and Nop2 126,127. These snoRNPs and methylases seem to associate with and act on early pre-60S r-particles 53,128. Second, also the *trans*-acting factor Nsa2 129 has been shown to bind to rRNA near the immature PTC, most specifically to positions close to the base of helix H89 in 25S rRNA 130, where it interacts with Nog1 and Nog2 40. Interestingly, Nsa2 associates with pre-60S r-particles only after formation of 27SB pre-rRNAs 53,131 and this association is dependent on the presence of other LSU maturation factors, including Nop2, Dbp10 and Nog1, on pre-60S r-particles 53. Third, it has been also shown that the RNA helicase Dbp10 binds around the base of helix H89 118. These sites partially overlap with those of Nsa2, Nmd3, and of GTPases Nug1 and Nog2 130,118,130. Indeed, Nug1 and Dbp10 functionally interact with each other 132. Moreover, the association of Dbp10 with pre-60S r-particles is dependent on the presence of Nug1 118.

All these findings, together with the above described binding sites of Nmd3, Nog1, Nog2 or Sdo1, as well as the assembly position of r-proteins such as L10, provides an idea of the, still to be unveiled, complex arrangement of sequential actions and interactions of factors during the maturation of strategically relevant, functional sites of r-subunits (for a review, see also 124).

### Arx1 versus translation-associated factors that bind the ribosome exit tunnel.

Arx1 is a non-essential *trans*-acting factor that has the capability to bind to FG-repeat nucleoporins, thereby, functioning as a RanGTP-independent export factor of pre-60S r-particles 133,134,135. The human orthologue of yeast Arx1, EBP1, folds as a MetAP, an enzyme removing the N-terminal methionine from nascent polypeptides as they emerge from the exit tunnel of the ribosome 133. Given the close homology between EBP1 and Arx1, it was deduced that Arx1 also conserves the MetAP core fold. However, both EBP1 and Arx1 lack MetAP activity, as the critical residues of the methionine-binding pocket are different 133. The interpretation of the cryo-EM structures of either purified pre-60S r-particles containing Arx1 70 or *in vitro* reconstituted 60S r-subunits complexed with Arx1 69 is in full agreement with this deduction. In these models, Arx1 binds nearby to the outside part of the PET (Figures 6A and 6B) 69,70 in a position that seems to overlap with the one of MetAPs 136. Consistently with this, the rRNA residues contacted by Arx1, which were identified by CRAC analysis, are clustering at the outside of the ribosome exit tunnel 70. Moreover, either the addition of a GFP-tag to r-proteins L25 (uL23) or L35 (uL29), which both surround the outside of the PET, or the depletion of L35 significantly reduce the binding of Arx1 to pre-60S r-particles 67,137.

In conclusion, these findings indicate that Arx1 could act as a structural proofreader of the correct formation of the solvent-side part of the exit tunnel 138. The inefficient recruitment of Arx1 to eventually aberrant pre-60S r-particles may impair export of these particles to the cytoplasm, leading to their transient accumulation in the nucleus and inducing their rapid turnover. Arx1 may also represent a placeholder for those ribosome-associated factors that bind the outside of the tunnel during translation, such as the ribosome-associated complex, the nascent polypeptide-associated complex, the chaperones Ssb1/Ssb2 or the signal recognition particle (for a review see, 139). This activity could prevent the premature recruitment of all these factors. The presence of Arx1 at its binding site also impairs the association of pre-60S r-particles with the endoplasmic reticulum-translocon complex, which acts as a channel to deliver nascent proteins to the lumen of the endoplasmic reticulum (e.g. see 140).

## PARAPHERNALIA FOR THE NUCLEAR IMPORT AND ASSEMBLY OF RIBOSOMAL PROTEINS

Most newly synthesized r-proteins need to be transported to the nucle(ol)us to reach their assembly sites in the pre-ribosomal particles. The specific interactions of the r-proteins with the general import factors, mostly β-karyopherins 141,142, are normally mutually exclusive with their interactions with the rRNAs. This feature also applies for the interactions that selected r-proteins undergo with specific factors that help their import or assembly, also referred as dedicated chaperones and escortins 143,144,145. In addition to help import or assembly, β-karyopherins, dedicated chaperones and escortins prevent the aggregation of r-proteins, which are prone to aggregate since they contain highly basic and intrinsically disordered extensions 146; these factors also impede r-proteins to either be degraded 147 or inappropriately interact with other cellular RNAs prior to their assembly into pre-ribosomal particles 146.

In a recent report, it has been shown that most, if not all, NLSs of yeast r-proteins reside within long non-globular extensions of the proteins. These extensions thread across the surface of the r-subunits making extensive contacts with the rRNAs or penetrate into the interior of the r-subunits intertwining with and stabilizing rRNAs 141. Kap123 is the RanGTP-dependent β-karyopherin in charge of recognising the NLSs of most r-proteins 142, although other β-karyopherins, such as Kap104, Kap108 or Kap121 143,148,149, and even the importin-α Kap60 150 have been reported to bind the NLSs of specific r-proteins. To our knowledge, no structural data of any r-protein bound to a karyopherin are available at atomic resolution, except for the recent determination of the crystal structure of Kap104 in complex with the PY-NLS of L4 (uL4) 149; here, the residues of the PY-NLS of L4 engages the concave surface on the structure of Kap104, similarly as other importins recognize their selected NLSs 145.

So far, seven specific systems, composed of a dedicated chaperone or an escortin and an r-protein, have been reported in yeast, a list that may still be far from being completed: Acl4•L4, Bcp1•L23, Rrb1•L3, Sqt1•L10, Syo1•L5•L11, Tsr2•S26 and Yar1•S3 (for a review, see 151). For most of them, the binding sites of the chaperone on the respective r-protein have been mapped, and for several of them structural information is also available. From all these studies, it could also be concluded that the mode of interaction of the respective r-proteins with their specific chaperone partners is highly similar and mutually exclusive with that observed for these r-proteins when assembled into the pre-ribosomal particles. Interestingly, most of these chaperones capture co-translational their targets by, in most cases, interacting with their very N-terminal ends, a property that minimizes the risk of r-protein aggregation 152.

## Acl4 and L4

Acl4 is a non-essential tetratricopeptide repeat-like (TPR-like) factor, which has been identified as the specific dedicated chaperone of free L4 (uL4) 153,154. Acl4 constitutes one exception to the principle of co-translationally recruitment through the N-terminal end of the r-protein partner since, although it is true that Acl4 is recruited to nascent L4, it directly interacts with the long internal loop of the r-protein (amino acids 43-114)^149^ ^153^,^154^. Additionally, the eukaryote-specific C-terminal extension of L4 harbours a PY-NLS, which binds specifically to another Acl4 molecule that is later replaced by the karyopherin Kap104 149,153,154. Importantly, both the internal loop and the C-terminal tail of L4 occupy strategic positions in mature LSUs 44,47; the internal loop penetrates into the core of the LSU in the direction to the PTC, forming a narrow constriction within the exit tunnel where it even forms some contacts with various residues of the nascent protein chains 155; the C-terminal tail of L4 threads a long tour across the solvent-exposed interface of the LSU in direction of the P-stalk, approaching and making numerous contacts with other r-proteins, such as L18 (eL18), L7 (uL30), L20 (eL20) and L21 (eL21) and rRNA sequences, including the expansion segments ES15 and ES7 of the 25S rRNA 44,156. Recently, the crystal structure of L4, excluding its C-terminal extension, in complex with Acl4 has been resolved at atomic resolution 149. In this complex, the majority of the internal loop of L4 is bound by the concave surface of Acl4, an interaction that is totally mutually exclusive with that this region of L4 forms within the LSUs 149.

It has been shown that Acl4 actively travels as a trimeric Kap104•L4•Acl4 from the cytoplasm to the nucle(ol)us where the assembly of the r-protein in early pre-60S r-particles takes place 79,152. As for other specific chaperones, Acl4 does not significantly associate with pre-60S r-particles 153,154, but is expected to facilitate the assembly of L4 into these particles; how this process mechanistically occurs is currently unclear.

## Rrb1 and L3

Rrb1 is WD-repeat protein predicted to form a β-propeller structure and involved in LSU maturation 157,158. The function of Rrb1 in LSU biogenesis is linked to that of the r-protein L3 (uL3) 157,158. Indeed, the Kressler laboratory has nicely demonstrated that Rrb1 captures nascent L3 in a co-translational manner via its interaction with the first 15 amino acids of the r-protein 152. Following this cytoplasmic recognition, Rrb1 then accompanies L3 to its assembly site on early nucleolar pre-60S r-particles 78,152,157. Unfortunately, no structural data have so far been reported for this interaction, although it has been speculated to occur in a similar manner as that between Sqt1, another WD-repeat protein, and its specific partner, the r-protein L10 (see below) 151. Despite this issue, it is clear that the interaction of L3 with Rrb1 is mutually exclusive with the ribosomal interaction of L3. In this sense, the N-terminal end of L3, which is recognized by Rrb1, penetrates deep into the core of the LSU where it reaches towards the PTC, nested between helices H90 and H92 of 25S rRNA 44,159.

The assembly path of L3 is totally unknown. Given the specific interaction of both L3 and Rrb1 with the members of the so-called the Dbp6-containing subcomplex (see 160), it is expected that the transfer of L3 from Rrb1 to its assembly site in early pre-60S r-particles could be facilitated by this subcomplex.

## Sqt1 and L10

The essential WD-repeat protein Sqt1 is a dedicated chaperone that has been proposed to facilitate the assembly of L10 (uL16) into cytoplasmic pre-60S r-particles 110,152,161. Two decades ago, Sqt1 was identified as a high-copy suppressor of selected *rpl10* mutants 161. Additionally, it has been reported that Sqt1 interacts with L10 by the two-hybrid system 161, by co-immunoprecipitation 110,152 or by an *in vitro* binding assay 152. Sqt1 recognizes the N-terminal part of L10 110,152,162 and, as described for Acl4 and Rrb1, the chaperone is already recruited to the nascent L10 as it is translated from its mRNA 152. The complex between the WD-repeat propeller-like domain of Sqt1 and the N-terminal part of L10 has been co-crystallized and structurally resolved to atomic resolution 152. This analysis shows how the very basic N-terminal end of L10, which forms an α-helix, is accommodated by the negatively charged top surface of the propeller 152. Importantly, this study also revealed that the conformation of the corresponding N-terminal residues of L10 bound to Sqt1 is not compatible, hence mutually exclusive, with the positioning of these residues, which interact with helix H89 of 25S rRNA, in the mature LSU. This information, together with all the genetic and biochemical data available for L10 and Sqt1, as well as those factors functionally related to L10, such as Nmd3, Sdo1 or Efl1, is very relevant to unravel the molecular dynamics of the delivery of L10 from Sqt1 to cytoplasmic pre-60S r-particles, which is still not fully understood (see 152 for further discussion of this issue).

## Syo1 and L5 and L11

**Figure 7:**
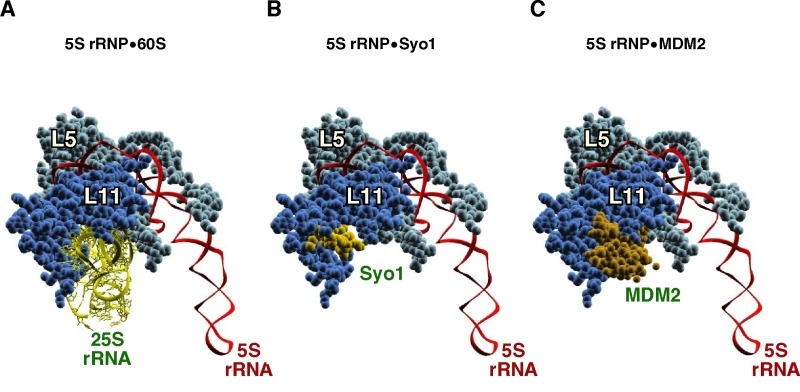
Figure 7: The helix H84 in the 60S r-subunit, the symportin Syo1 and the p53 regulator MDM2 share the same binding site on L11 r-protein. **(A) **Structure of the 5S RNP as is assembled in the mature 60S r-subunit bound to 25S rRNA (nucleotides 2651-2750 comprising helices H83, H84, H85 and H86). **(B) **Interaction of a specific region of Syo1, called Syo1-HS (amino acids 328-384), with L11 in the context of the 5S RNP. **(C) **Structure of the 5S RNP bound to a distinct fragment of MDM2 (amino acids 293-334). The fragment of 25S rRNA is coloured in yellow, that of Syo1 in light gold, and that of MDM2 in dark gold; 5S rRNA in highlighted in red, L5 in cyan and L11 in blue. PDB ID: The 5S RNP bound to 25S rRNA was taken from 3U5I and 3U5H 44; Syo1-HS fragment was extracted from 5AFF 163 after superimposing the structure shown here with that present in the PDB file 4GMN 144; MDM2 fragment was taken from 4XXP 164 after superimposing the structure shown in this file with that of L11 shown in A.

The symportin Syo1 is an exceptional factor since it is the only described case of a chaperone dedicated to the synchronous co-import of two conserved r-proteins to the nucle(ol)us: L5 (uL18) and L11 (uL5), which are the two protein components of the 5S rRNP structure of LSUs 144. The crystal structure of *C. thermophilum* Syo1 complexes reconstituted with L5, L11 and/or 5S rRNA have been reported 144,163; Syo1 is an open α-solenoid comprised of four consecutive ARM-repeats followed by six HEAT-repeats; notably, the acidic loop, connecting the two first HEAT repeats long, harbours a critical helical segment, known as Syo1-HS, for function. While the ARM-repeat domain is not involved in cargo binding, the HEAT-repeat domain can simultaneously accommodate both L5 and L11 on opposite sides 144. More specifically, the eukaryote-specific N-terminal end of L5 binds a groove formed at the inner surface by the four first HEAT-repeats of Syo1 144, while L11, which adopts a hand-like shape, primarily interacts via the internal concave β-sheets from its "palm" with the Syo1-HS segment; other contacts between Syo1 and L11 are made between a particular loop located at the "knuckles" of the r-protein and the outer side of Syo1's HEAT repeats 1 to 3 (for details, see 163). Strikingly, the N-terminal end of L5 also contributes to 5S rRNA binding 44,47 and the binding of L11 to the Syo1-HS is basically identical to and mutually exclusive with the interaction with the helix H84 of the 25S rRNA (Figures 7A and 7B) in both pre-60S r-particles 41 and mature LSUs 44,47.

The intensive work performed on Syo1, together with the significant amount of genetic, biochemical and structural data available on the formation of the 5S RNP complex, has allowed to establish the following model for the assembly of this complex into early pre-60S r-particles: (i) first, Syo1 captures nascent L5, in this manner, preventing L5 to misfold and aggregate 152. Binding of L11 to Syo1 also apparently occurs in the cytoplasm but not in a co-translational manner 144,154. (ii) The trimeric Syo1•L5•L11 complex is then recognized, via the N-terminal PY-NLS of Syo1, by the importin Kap104, and escorted to the site of assembly in the nucle(ol)us 144. (iii) Upon nuclear arrival, RanGTP promotes the release of Kap104 and, concomitantly, the 5S rRNA likely binds to the trimeric complex 144. Structural data suggest that binding of 5S rRNA induces a change in the conformation of both r-proteins within the Syo1•L5•L11 complex 163. (iv) Now, the pre-5S RNP is likely ready for incorporation into early pre-60S r-particles 41,165, a process that is facilitated by the assembly factors Rpf2 and Rrs1 166. Structural and functional analyses strongly suggest that the Rpf2•Rrs1 complex can bind the Syo1•5S rRNA•L5•L11 complex, by this way recruiting it to nascent pre-ribosomal particles 167,168,169; however, Syo1 is never found associated to pre-ribosomal particles, thus, the interaction of Rpf2•Rrs1 complex with 5S RNP in the context of pre-60S r-particles must somehow induce the recycling of the symportin 167,169. Interestingly, the recruitment of 5S RNP to particles includes the docking of L11 to helix H84 of 25S rRNA, while keeping the rest of the RNP in an almost 180° rotated configuration that is different from the one adopted in mature 60S r-subunits 41,167,169. Since the Rpf2•Rrs1 complex (and Rsa4) interacts with the 5S RNP in this initial conformation but is unable to do it in the rotated, final conformation in mature LSUs, it has been suggested that the Rfp2•Rrs1 complex defines the timing for assembly of 5S RNP into distinct pre-60S pre-ribosomal particles, thereby exerting a quality control surveillance for this important step during the formation of LSUs 130,169.

## Escortins of r-proteins: Tsr2 for S26, and Bcp1 for L23

Another factor involved in the correct assembly of a specific r-protein is Tsr2. This factor was first identified as a non-essential protein required for 20S pre-rRNA maturation, and hence for SSU biogenesis 170. More specifically, Tsr2 has been described to be required for cytoplasmic processing of 20S pre-rRNA to mature 18S rRNA 143. This role of Tsr2 is clearly linked to its specific association with the r-protein S26 (eS26) 143,170. Indeed, the Panse laboratory has shown that S26 binds directly to Tsr2 and is also required for cytoplasmic processing of 20S pre-rRNA 143. In clear contrast to other dedicated chaperones, Tsr2 only interacts with S26 once both proteins have been independently imported to the nucleus by selected karyopherins, such as Kap123 143. Biochemical evidence suggests that Tsr2 has the capacity to extract S26 from its karyopherin by a RanGTP-independent mechanism 143. The Tsr2•S26 interaction impedes the aggregation of S26, while presumably permitting the escort of the r-protein to its correct assembly site on early 90S pre-ribosomal particles. However, Tsr2 does not significantly interact with the nascent pre-ribosomal particles 143. Due to this role, Tsr2 has been proposed to be an "escortin", which refers to any *trans*-acting factor able to release distinct r-proteins from their specific import systems and transfer them to the respective nascent pre-ribosomal particles where they assemble 143. Further exciting experiments are clearly required to precisely understand how exactly Tsr2 and S26 interact with each other, and whether the Tsr2-bound state is mutually exclusive with that of S26 within the SSU. As discussed in the next section, S26 once dissociated from Tsr2, binds r-protein S14 to form a trimeric Fap7•S14•S26 complex (171; see later).

Bcp1 is an essential factor required for proper LSU maturation 172. Recently, it has been suggested that Bcp1 works as a chaperone of L23 (uL14) 173. More specifically, Bcp1 seems to work as an escortin to dissociate nuclear and non-ribosome bound L23 from any of the distinct karyopherins, Kap123 or Kap121, involved in its nuclear-cytoplasmic import. In the nucleus, Bcp1 appears to guide the assembly of L23 in early pre-60S r-particles. Similarly to what has been previously reported for Tsr2, the exchange of L23 from its karyopherin to Bcp1 is also likely RanGTP-independent 173. Unfortunately, as for Tsr2, neither structural data nor information on the binding site of Bcp1 on L23 has so far been reported. These issues are of special interest to understand how L23 is delivered from Bcp1 to pre-60S r-particles and to get insight into its relation with *trans*-acting factors such as Tif6, whose binding site mainly involves L23 47,116,123.

## Yar1 and S3

The last member of this as yet likely incomplete inventory of factors is the ankyrin-repeat factor Yar1, which directly interacts with the free r-protein S3 (uS3) 174. S3 consists of two well-defined globular domains, one N-terminal and another C-terminal, preceded and followed by unstructured terminal extensions, respectively 175. As expertly envisioned by D. Lycan about a decade ago 174, Yar1 works as a molecular chaperone to keep S3 protected from *in vivo* agregation 176.

Indeed, as described for many of the above dedicated chaperones, Yar1 directly interacts with free S3 by capturing its N-terminal unstructured domain in a co-translational manner 152. Then, Yar1 accompanies S3 from the cytoplasm to the nucleus where it seems to assist the proper assembly of S3 into late pre-40S r-particles 176,177. The detailed characterization done for Yar1 has suggested the following scenario for the stable assembly of S3: (i) Yar1 co-translationally binds the N-terminal domain of S3, particularly the N-terminal α-helix of S3, by a concave pocket that is formed by its four ankyrin repeats, while its fourth ankyrin-repeat mediates contacts with the C-terminal domain of S3 152,178,179. (ii) Importantly, it has been shown that cytoplasmic S3 dimerizes through its C-terminal globular domain *in vitro* and *in vivo* 178. Curiously, *in vivo*, only one S3 N-terminal domain of the dimer is associated with Yar1, while the second one is bound by the importin-α Kap60/Srp1 150. Kap60 binds the very N-terminal monopartite NLS of S3, most likely through its major binding site for cargos, and then to importin-α Kap95 through its orthodoxal IBB domain 150; the S3 NLS is located contiguous to the Yar1-binding site, however, it has been established that the binding of Yar1 and Kap60 to one N-terminal domain of S3 is mutually exclusive, suggesting that one Kap60 molecule rapidly replaces only one Yar1 protein in the (Yar1•S3)2 tetrameric complex 150. (iii) This asymmetric Kap95•Kap60•S3•S3•Yar1 complex is actively imported into the nucleus, where rapid RanGTP-dependent dissociation of the importer system presumably occurs. Other importins have been proposed to be redundantly able to mediate the import of S3, including Kap123 150. (iv) How exactly the transition from Yar1-bound S3 to pre-40S r-particle-bound S3 occurs is unclear; it is worth to mention that these two states are incompatible with each other, since the N-terminal domain of S3, which binds Kap60 and Yar1, is in contact, with several neighbouring r-proteins, such as S10 (eS10), S20 (uS10) and S29 (uS14), and specific rRNA residues of the 18S rRNA helix H41 within mature SSUs 44,91,141,178. Moreover, the N-terminal domain of S3 in the S3•Yar1 complex is rotated ca. 180° relative to the C-terminal domain compared to its configuration in mature SSUs, and therefore, away from its contact surfaces in SSUs. Additionally, the dimerization of S3 masks the SSU rRNA-binding site of the C-terminal domain 179. Initial assembly of S3 within pre-40S r-particles is suspected to occur through the C-terminal domain. This fact implies that the second copy of S3 from the tetramer is released as soon as the assembly of one molecule of S3 initiates and likely assembles in parallel within another orphan pre-40S r-particle. Concomitantly, the remaining Yar1 molecule dissociates and is replaced by the *trans*-acting factor Ltv1, which seems to have higher affinity for S3 179,180. Ltv1 and Yar1 have partially overlapping binding sites within the N-terminal domain of S3, as demonstrated *in vitro* 179. This result is consistent with the reported cryo-EM position of Ltv1 on pre-40S r-particles 21, and the sites of interaction of Ltv1, described by the CRAC methodology, within helices H41 and H16 of the 18S rRNA, which are located at the head and the shoulder of the SSU, respectively 103. Importantly, different evidence indicates that, although S3 is present in late pre-40S r-particles containing Ltv1, it is still not bound in its final conformation 21,101,177,181. Thus, it can be extracted from these particles in the presence of a high salt concentration as a complex with Ltv1 and another 40S assembly factor, Enp1 101. Presumably, in pre-40S r-particles, S3 is associated via its C-terminal domain at a position close to its final binding site 180. However, its N-terminal domain is likely still in the rotated conformation it adopts when bound to Yar1, while it probably interacts similarly to the central region of Ltv1 179,182. (v) The stable incorporation of S3 must occur concomitantly to the release of Enp1 and Ltv1 from cytoplasmic pre-40S r-particles, which is dependent on the phosphorylation of at least Ltv1 at selected serine residues by the kinase Hrr25 (human casein kinase1 δ or 1ε) 101,179,180. It has been postulated that the release of Ltv1 from pre-40S r-particles allows the N-terminal domain of S3 to assemble into its mature binding site within the context of S10, S20 and S29 and 18S rRNA residues of helix H41 179,180. Indeed, the interaction of the C-terminal part of Ltv1 with pre-40S r-particles seems to be incompatible with the positioning of S3 and the presence of S10 in these particles 21,103,177. The C-terminal domain of S3 also changes its conformation during the cytoplasmic maturation of SSUs so that it acquires its final position by stably interacting with r-proteins Asc1, which is the yeast orthologue of mammal RACK1, and S17 (eS17) (for experimental evidence, see references 21,177). More recently, refined cryo-EM analysis of late/cytoplasmic pre-40S r-particles confirmed that S3 is still not bound at its mature site in these precursor particles, however, this study has questioned this particular model, involving the repositioning of the globular N- and C-terminal domains of S3 183. Nevertheless, given the intrinsic dynamic nature of the maturation pathway of pre-ribosomal subunits, it could also be feasible that these precursors had already undergone many of the conformational changes, involving S3 and Ltv1 that have been suggested to occur. Moreover, whether these structural rearrangements end with the release of Enp1 and Ltv1 also needs further clarification.

**Table 1:** Examples of placeholding situations during the ribosome biogenesis pathway. ^1^ A question mark indicates that the placeholder correspondence or the mutually exclusive interaction is suspected and has not been experimentally addressed.

**Factor**	**Counterpart ^1^**	**Reference**
**Ribosomal-like proteins**		
Mrt4	Mex67, P0 (uL10)	20 45 46
Rlp24	L24 (eL24)	28
***Trans*-acting factors**		
Arx1	EBP1, RAC, NAC, Ssb1/2, Sec61 complex	69 70
Bms1	Tsr1 (?)	95 99
Dbp10	Nmd3	118
Dim2	Krr1 (?)	189
Efl1	EF-2 (Eft1/Eft2), Tif6	19 116
Nmd3	eIF5A	43 114
Nog1	Rei1	40
Nog2/Nug2	Nmd3	40 115
Nsa2	Nmd3	40
Nug1	Nmd3	118
Rei1	Reh1	43 68
Sdo1	Nmd3	114 116
Tsr1	eIF5B, Rio1	21 102
**Dedicated chaperones and escortins**		
Acl4	L4 (uL4)	149 153 154
Bcp1	L23 (uL14)	173
Fap7	S14 (uS11)	171 184 185
Rrb1	L3 (uL3)	152 157 158
Sqt1	L10 (uL16)	152 162
Syo1	L5 (uL18), L11 (uL5)	144 163
Tsr2	S26 (eS26)	143 170 171
Yar1	S3 (uS3)	150 152 174 178 179
**Box H/ACA snoRNP assembly factors**		
Naf1	Gar1	188
Shq1	H/ACA snoRNA	186 187

## PLACEHOLDING BY MOLECULAR MIMICRY

The term "Protein-RNA mimicry" applies to the capability of a protein (or a protein domain) to imitate the structure of a distinct domain of an RNA that normally binds to a different RNA or protein. The interaction of the latter molecule with either its natural partner or the mimicking protein is normally used as a control step for the correct function of the biological process where this molecule participates (for a review, see 190). Few cases of molecular mimicry have been reported related to the ribosome; the most classical one groups those translation factors that mimic tRNAs and that bind to the ribosome similarly as tRNAs do 190. The pathway of ribosome assembly has also taken advantage from using the protein-RNA mimicry concept. One of these examples, as already mentioned above, represents the Syo1-HS domain that imitates the 25S rRNA helix H84, which seems to control the timing of assembly of L11 into pre-60S r-particles 163. Strikingly, the same mimicry principle has been exploited for the activation of p53 during the mammalian nucleolar stress response, which is triggered by sequestering the E3 ubiquitin ligase MDM2 through its complex with 5S RNP (reviewed in 191). Thus, recent structural work has shown that, similarly to Syo1, a particular domain of MDM2 mimics the 25S rRNA binding to L11 so that the interaction of 5S rRNP to MDM2 or to the LSU are also mutually exclusive (Figures 7A and 7C) 164. In this section, we review on other biological relevant examples of mimicry on ribosome assembly (see also, 192).

### Fap7 and S14

Fap7 is a conserved essential ATPase required for SSU formation, more specifically for the cytoplasmic maturation of 20S pre-rRNA to 18S rRNA 170,193. This function is linked to that of r-protein S14 (uS11) and coordinated with that of other late SSU assembly factors (namely, Dim1, Enp1, Nob1, Pno1 and Tsr1); indeed, mutations in the eukaryote-specific C-terminal tail of S14 block processing of 20S pre-rRNA to mature 18S rRNA similarly as the depletion of Fap7 194; moreover, those late SSU assembly factors persisted in 80S-like particles in the absence of Fap7 104.

It has been shown that Fap7 directly and stably interacts with S14 *in vitro* 193,195 and *in vivo* 184,195 but only weakly or transiently with pre-40S r-particles 104,171,193,195. The structure of the Fap7•S14 heterodimer has been solved. In this complex, the interaction of Fap7 with S14 blocks the RNA binding surface of S14 184,185, hence, the binding of Fap7 to S14 clearly competes with the positioning of S14 in its final conformation in mature or almost mature SSUs. Interestingly, the structural studies indicate that indeed, Fap7 acts as an RNA mimic, using protein side chains to reproduce specific contacts of the 18S rRNA with S14 185. Physiologically, this interaction which is regulated through the ATP binding and hydrolysis activities of Fap7 (for further details, see 184,185), suggests that Fap7 acts as a dedicated and enzymatic chaperone for S14. In this way, Fap7 might protect S14 from aggregation and/or nonspecific interaction with other RNAs and regulate the correct timing of S14 assembly into pre-40S r-particles. In agreement with this, recombinant S14 from *E. coli* showed poor solubility unless it is co-expressed with Fap7 185; moreover, depletion of Fap7 causes a strong decrease in the *in vivo* protein levels of S14 in *S. cerevisiae* 171. However, there is so far no evidence for co-translational capturing of S14 by Fap7 (discussed in 151).

Although different scenarios have been proposed, the mode of S14 release from Fap7 and the mechanism of its concomitant incorporation into pre-40S r-subunits are still unclear 171,184,185. Interestingly, the Panse laboratory has recently shown that the assembly of S14 and S26 into pre-40S r-particles is interdependent 171. Fap7 promotes the formation of a ternary complex containing both S14 and S26 171, which are neighbouring proteins that directly interact in the mature SSU 44,91. In the Fap7-containing complex, S26 and S14 might interact with each other similarly as they do in the context of the mature SSU 171. Thus, it was concluded that Fap7 is an example of a factor enabling nucleation a module of two r-proteins, which then assemble en bloc into relatively early pre-40S r-particles 171.

### Shq1, Naf1 and the formation of H/ACA snoRNPs

Box H/ACA ribonucleoparticles (snoRNPs) are a family of small RNA-protein complexes conserved in archaea and eukaryotes that convert uridine into pseudouridine at specific sites within rRNAs and snRNAs, mediate early pre-rRNA processing reactions and r-subunit assembly and maintain telomerase stability (reviewed in 87,196). It has been proposed that all mature H/ACA snoRNPs are composed of a distinct box H/ACA snoRNA, which selectively base pairs with its target RNA, and two sets of four conserved core proteins, one per each hairpin motif of the bipartite structure of the snoRNA: the pseudouridinase Cbf5 (NAP57 in rodents and dyskerin in humans), Nop10, Nhp2 (L7Ae in archaea) and Gar1 87,196,197. Although these complexes can self-assemble *in vitro* (for a reference, see 198), H/ACA snoRNP formation requires multiples *trans*-acting factors *in vivo* 197. Among these factors, two conserved and essential proteins, Shq1 and Naf1, are specifically required for the stable accumulation of all box H/ACA snoRNAs, without being part of the mature particles in either the nucleolus or the Cajal bodies (e.g. 199,200,201, reviewed in 197).

It has been shown that Shq1 functions in an early step of H/ACA snoRNP biogenesis 202,203. Shq1 apparently binds newly synthesized Cbf5 acting as a chaperone that prevents its misfolding, aggregation and degradation. Additionally, Shq1 prevents non-specific interactions of nascent Cbf5 with other RNAs before its association with box H/ACA snoRNAs 203. The Shq1•Cbf5 interaction mainly occurs through the central and Shq1-specific C-terminal (SSD) domains of Shq1 and the RNA-binding interface of Cbf5 203. Indeed, it has been shown that Shq1 acts an RNA mimic; specific residues of Shq1 occupy the same position on nascent Cbf5 as selected RNA residues of an H/ACA snoRNA do within mature H/ACA snoRNPs 186,187. As a corollary, the binding of Cbf5 to either Shq1 or an H/ACA snoRNA is obligatorily mutually exclusive. The subsequent release of Shq1 by a specific chaperone complex, R2TP, allows the progression of H/ACA snoRNP biogenesis. Moreover, it has been proposed that another assembly factor, known as Naf1, brings Cbf5, Nop10 and Nhp2 to nascent box H/ACA RNAs at their site of transcription 196,197.

Naf1 was identified as a nuclear factor able to interact with both the C-terminal domain of RNA polymerase II and nascent H/ACA snoRNAs 200. At this time, a certain structural homology between Naf1 and the core domain of Gar1 was predicted 200, thus, being suggestive of a placeholder activity of Naf1 for Gar1. In agreement with this hypothesis, human NAF1 and GAR1 bind NAP57 (human Cbf5) competitively and in a mutually exclusive manner 204. Moreover, crystallography demonstrated the structural homology between yeast Naf1 and the protein domain that in archaeal Gar1 binds Cbf5 188. Importantly, Naf1-containing nascent H/ACA snoRNPs seem to be fully inactive regarding pseudouridinase activity; only the later replacement of Naf1 by Gar1 during H/ACA snoRNP biogenesis permits the formation of an active snoRNP 198,204. This latter result strongly suggests that the physiological function of Naf1, as the one of a placeholder factor, is keeping the emerging RNA-based machine inactive in order to prevent undesired effects on non-cognate substrates.

### FUTURE PERSPECTIVES AND CONCLUSIONS

In this review, we have described a relatively large number of *trans*-acting assembly factors that work as placeholder factors during the ribosome biogenesis process. The placeholder hypothesis, which was initially suggested as the functional corollary of the presence of a few assembly factors showing a high degree of similarity to distinct r-proteins over their entire length 31, has been confirmed for more *trans*-acting factors than initially envisaged. This pleasant surprise has come as the experimental consequence of the combination of powerful biochemical, cell biological and genetic studies with the structural characterization of pre-ribosomal particles or reconstituted r-subunits by X-ray crystallography and cryo-EM from sub- to near-atomic resolution (e.g. 40). This new mode to approach the pathway of ribosome biogenesis is providing for the first time clues on how *trans*-acting factors bind and operate to accommodate other factors or r-proteins during the maturation reactions of the nascent r-subunits (for a review, see 124).

The phenotypic analyses of loss-of-function mutant variants of placeholder factors has proven to be very useful for revealing their functional relevance during the eukaryotic ribosome assembly process and have provided insights on why these factors have been positively selected during evolution. We have discussed several functions for placeholder factors: (i) Some placeholder factors (e.g. Nog2 or Mrt4) clearly control the position and timing of association or assembly of their counterparts. In the absence of these placeholder factors, the respective counterparts are prematurely recruited to the nascent pre-ribosomal particles, impeding important events that only the placeholder factors are able to exert. We can envisage a scenario where these placeholder factors are guarding key sites in premature r-particles from the binding of other factors or r-proteins. The premature recruitment of these later-associating proteins could even generate kinetically trapped assembly intermediates that impede downstream maturation steps. However, in other cases (e.g. Rlp24), although the function of the placeholder factor has been shown essential for the correct progression of the maturation of the r-subunit, it is still unclear whether a direct functional relationship exists between the placeholder factor and its counterpart r-protein. (ii) Many of the strategies involving placeholder factors during ribosome biogenesis ensure that the nascent r-particles follow a step-dependent and ordered process of assembly. These steps are unidirectional and therefore irreversible. For example, nuclear pre-60S r-particles are unable to acquire export competence until Nog2 is replaced by Nmd3; cytoplasmic maturation of pre-60S r-particles does not properly proceed if Nog1 is not efficiently released and replaced by Rei1; Tsr1 delays association of either eIF5B or Rio1 with cytoplasmic pre-40S r-particles. (iii) Specific dedicated chaperones and/or escortins carry out mutually exclusive interactions with other factors, rRNA or r-proteins. As discussed herein, these factors assist the import and assembly of distinct r-proteins while preventing their intrinsic tendency to aggregate, their degradation and/or their non-specific association to non-cognate substrates. In some cases, these chaperones act as rRNA mimics when they exert their placeholding activity. By mimicking, chaperones and escortins are even able to guarantee the proper folding of their substrates. As also discussed, these factors are even able to promote the interdependent assembly of more than one r-protein at the same time ensuring stoichiometry. (iv) A considerable group of placeholder factors might have critical roles in structural proofreading, as previously discussed by A. W. Johnson 138. A mechanism based on structural proofreading implies that the binding of a distinct factor or r-proteins depends on the proper generation of a specific site only after completion of selected upstream assembly steps. This strategy is used during export and cytoplasmic maturation of r-subunits, provides tools and mechanisms to detect assembly errors and ensures the specific cytoplasmic assembly of the last r-proteins. Equally important are strategies based on functional proofreading (discussed in 55,122) where key functional centres in r-subunits are inspected by a considerable number of factors, such as Arx1, which examines the solvent-exposed exit side of the PET, Nog1, Rei1, Reh1 and Sdo1, which probe the length and integrity of the PET, Nmd3, which examines the E- and P-sites of nascent LSUs, Efl1, which together with Sdo1 inspects the integrity of the P-site and the GAC regions of nascent LSUs, the P-stalk protecting Mrt4, etc. This functional proofreading strategy ensures that only properly assembled nascent r-particles undergo final maturation while simultaneously preventing premature translation by masking the active sites of the ribosome.

There is plenty more work ahead to fully understand the exact function of most, if not all, placeholder factors and the exact relationship with their counterparts during ribosome assembly. Further work is required to acquire more data on the binding sites and complete 3D maps at high resolution of r-particles containing many of the placeholder factors so far known. These aspects are essential to obtain precise information on the location of the placeholder factors relative to the pre-rRNAs, other factors and r-proteins. For many of them, their structures are still unsolved (e.g. Drg1, Mex67, Nug2). This also applies to some of the dedicated chaperones and escortins so far identified (e.g. Bcp1, Rrb1, Tsr2). Most importantly, the exact sequence of the mechanistic events that lead to the exchange of a placeholder by its counterpart also remains to be dissected for many of the examples described herein.

Finally, we would like to remark that mutations in several placeholder factors have been linked to human diseases. (i) These include the Shwachman-Diamond Syndrome, where about 90% of the patients have mutations in the SBDS gene. These mutant variants have been shown to be defective in the release of Tif6 from cytoplasmic pre-60S r-particles (see, 116,205, and reference therein). (ii) Moreover, mutations in the *RPL10* gene have been identified in patients of T-cell acute lymphoblastic leukaemia and they also seem to impair the efficient release of Tif6 and Nmd3 from cytoplasmic pre-60S r-particles (see 206, and references therein). (iii) The Syndrome 5q- seems to be caused by a RPS14 haploinsuficiency (reviewed in 207), thus, it is reasonable to imagine that certain loss-of-function mutations in FAP7 could be identified in the future as linked to this disease, in a similar manner as mutations in RPS26 or its escortin TSR2 have been linked to the Diamond-Blackfan Anemia (discussed in 151). (iv) Some mutations in dyskerin (human Cbf5) related to X-linked dyskeratosis congenita lead to the destabilization of the interaction of the mutant variant of dyskerin with its placeholder SHQ1 187. (v) Finally, mutually exclusive binding of MDM2 and 25S rRNA to the 5S RNP is the basis of p53 activation and signalling in numerous pathophysiological situations (discussed in 191). In conclusion, both the academic and the biomedical fields could benefit of the surely attractive and productive research on placeholder situations during ribosome biogenesis in the coming years.

## SUPPLEMENTAL MATERIAL

Click here for supplemental data file.

All supplemental data for this article are also available online at http://microbialcell.com/researcharticles/placeholder-factors-in-ribosome-biogenesis-please-pave-my-way/.
