# Supplementary material for: Placeholder factors in ribosome biogenesis: please, pave my way
Source: Microb Cell. 2017 Apr 27;4(5):144–68. doi: 10.15698/mic2017.05.572 (PMC5425277; doi:10.15698/mic2017.05.572)
Supplement: Supplementary file 1 [file mic-04-144-s01.pdf]

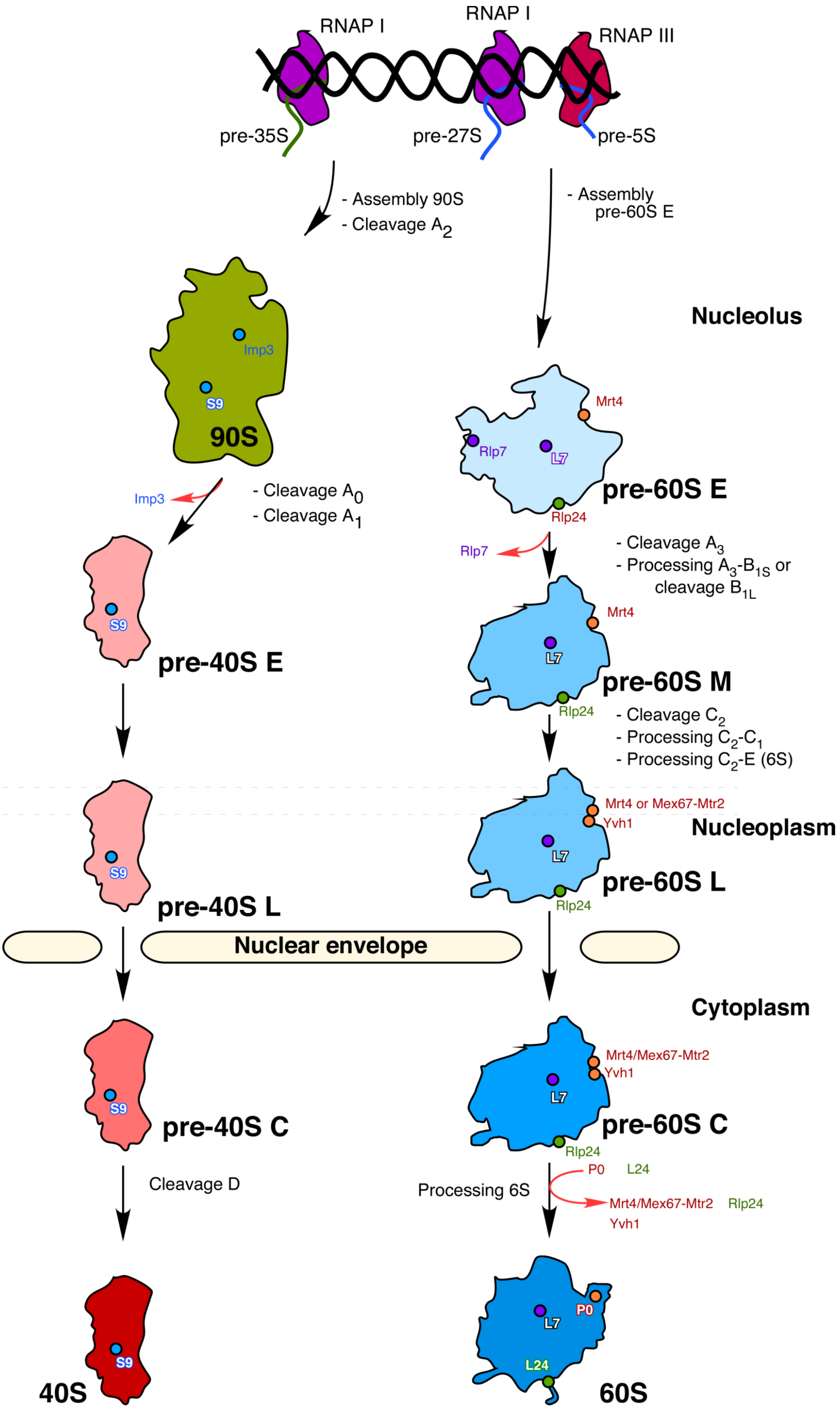

RNAP I
RNAP I
RNAP III
pre-35S
pre-27S
pre-5S
- Assembly 90S
- Cleavage A_2
- Assembly pre-60S E
Nucleolus
Imp3
S9
90S
Mrt4
Rlp7
L7
pre-60S E
Rlp24
Imp3
- Cleavage A_0
- Cleavage A_1
Rlp7
- Cleavage A_3
- Processing A_3-B_{1S} or cleavage B_{1L}
S9
pre-40S E
Mrt4
L7
pre-60S M
Rlp24
- Cleavage C_2
- Processing C_2-C_1
- Processing C_2-E (6S)
S9
Mrt4 or Mex67-Mtr2
Yvh1
Nucleoplasm
L7
pre-60S L
pre-40S L
Rlp24
Nuclear envelope
Cytoplasm
S9
pre-40S C
Mrt4/Mex67-Mtr2
Yvh1
L7
pre-60S C
Rlp24
Cleavage D
Processing 6S
P0
L24
Mrt4/Mex67-Mtr2
Rlp24
Yvh1
S9
40S
L7
P0
L24
60S

SUPPLEMENTARY FIGURE S1: Current model for the ribosome biogenesis pathway from *S. cerevisiae*. The different predicted particles along the synthesis of both SSUs and LSUs are depicted. The first detected particle is called 90S pre-ribosomal particle or SSU processome. This particle is nucleolar and contains either the nascent pre-rRNA, which has been cleaved at site A_2 in the co-transcriptional pre-rRNA processing branch, or 35S pre-rRNA in the post-transcriptional processing branch (not shown). This pre-rRNA is transcribed by the RNA polymerase I (RNAP I), while pre-5S is transcribed by RNAP III. 90S particles contain about three dozens of *trans*-acting factors and many r-proteins from the SSU. Maturation of the 90S particle involves the dissociation of the early pre-40S particle from the bulk of these factors, except few of them. Pre-40S particles are termed, according to their position in the pathway, early (E), late (L) and cytoplasmic (C). Cleavage D occurs in the cytoplasm within a 80S-like complex that contains not only the pre-40S particle C but also a mature LSU (not shown). Pre-60S particles assemble on the remaining part of the nascent pre-rRNA transcript. Pre-60S particles are termed early (E), middle (M), late (L) and cytoplasmic (C). The pre-rRNA processing reactions that occur inside the particles as they travel from the nucleolus to the cytoplasm are depicted. About 20 placeholder *trans*-acting factors have been described so far. In the figure, we have highlighted the dynamics of association and dissociation of ribosomal-like factors and their r-protein counterparts. Note that only two factors, Mrt4 and Rlp24, harbour placeholding activity. See text for further details.

A

```
RPP0     1 ------MGGIREKKAEYFAKLREY---LEEYKSLFVVGVDNVSSQQMHEVRKELRGRAVV
MRT4    11 -TLAQTDKKGRENKERIFDEVREA---LDTYRYVWVLHLDDVRTPVLQEIRTSWAGSKLI
MEX67  275 DALGQSSTDFATNFLNLWDNNREQLLNLYSPQSQFSVSVDSTIPPSTVTDSDQTPAFGYY

RPP0    52 LMGKNTMVRRAI-----RGFLSDLPDFEKLLPFVKGNVGFVFTNEPLTEIKNVIVSNRVA
MRT4    67 -MGKRKVLQKALGEKREEEYKENLYQLSKL---CSGVTGLLFTDEDVNTVKEYFKSYVRS
MEX67  335 -MSSSRNISKVSSEKSIQQRLS-------------------IGQESINSIFKTLPKTKHH

RPP0   107 APARAGAVAP----------------------EDIWVRAVNTGMEPGKTSFFQALGVPTK
MRT4   123 DYSRPNTKAPLTFTIPE--GIVYSRGGQIPAEEDV---PMIHSLEPTMRNKFE---IPTK
MEX67  375 LQEQPNEYSMETISYPQINGFVITLHGFF---EETG-KPELESNKKTGKNNYQ---KNRR

RPP0   145 IARGTIEIVSDVKVVDAGNKVGQSEASLLNLLNISPFTFGLTVVQVYDNGQVFPSSILDI
MRT4   175 IKAGKITIDSPYLVCTEGEKLDVRQALILKQFGIAASEFKVKVSAYYDN----DSST---
MEX67  428 YNHGYNSTSNNKL---SKKSFDRTWVIVPMNNSVIIASDLLTVRA-YSTGAWKTASI---
```

B

```
RPL24A   1 MKVEIDSFSGAKIYPGRGTLFVRGDSKIFRFQNSKSASLFKQRKNPRRIAWTVLFRKHHK
RLP24    1 MRIYQCHFCSSPCYPGHGIMFVRNDAKEFRFCRSKCHKAFKQRRNPRKLKWTKAFRKAAG

RPL24A  61 KGITEEVA---KKRSRKTVKAQRPITGASLDLIKERRSLKPEVRKANREEKLKANKEK--
RLP24   61 KELAVDSTLTFAQRRNVPVRYNRELVATTLKAMARIEEIRQKRERAFYKNRMRGNKEKDF

RPL24A 116 -------------------KKAEKAARKAEKAKSAGTQSSKFSKQQ-----------AKG
RLP24  121 LRDKKLVESNPELLRIREVEIARKLAKEQERAESVSEQEESEEEEEDMEIDSDEEEEEQL

RPL24A 146 AFQKVAATSR---------
RLP24  181 EKQKILLKNRRRNTKKIAF
```

C

```
RPL7A    1 MAAEK------------IL--------------------TPESQLKKSKAQQ----KTAE
RLP7     1 MSSTQDSKAQTLNSNPEILLRKRRNADRTRIERQELAKKKREEQIKKKRSNKNKFVRAES

RPL7A   25 QVAAERAARKAANKEKRAIILERNAAYQKEYETAERNIIQAKRDAKAAGS----------
RLP7    61 IVAKTLATSREKERIKRVSILEDKKAKNETQHIASGKDFILKITEKANGAEENSVDLEET

RPL7A   75 ------------YYVEAQHKLVFVVRIKG--INKIPPKPRKVLQLLRLTRINSGTFVKVT
RLP7   121 EEEEDDGLIREKTTYDGKPALLFIVRVRGPLAVNIPNKAFKILSLLRLVETNTGVFVKLT

RPL7A  121 KATLELLKLIEPYVAYGYPSYSTIRQLVYKRGF------GKINKQRVPLSDNAIIEANLG
RLP7   181 KNVYPLLKVIAPYVVIGKPSLSSIRSLIQKRGRIIYKGENEAPHEIVLNDNNIVEEQLG

RPL7A  175 KYGILSIDDLIHEIITVGPHFKQANNFLWPFKLSNPSGGWGVPRKFKHFIQGGSFGNREE
RLP7   241 DHGIICVEDIIHEIATMGESFSVCNFFLQPFKLNREVSGFGSLNRLRKIKQREAESRTRQ

RPL7A  235 FIN------------KLVKSMN
RLP7   301 FSNAATAPVIEVDIDSLLAKLN
```

D

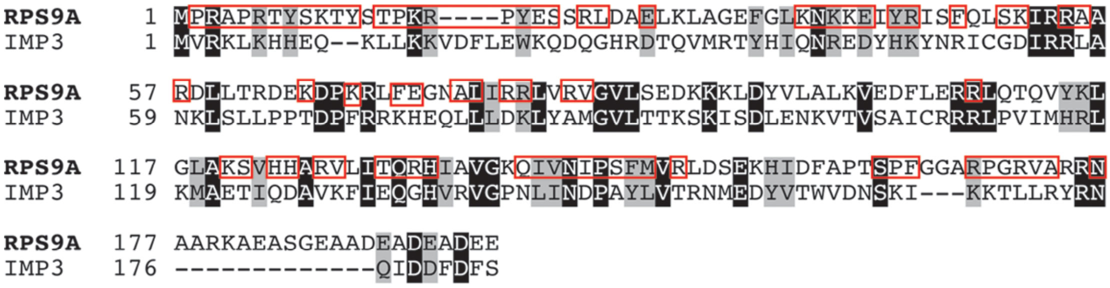

E

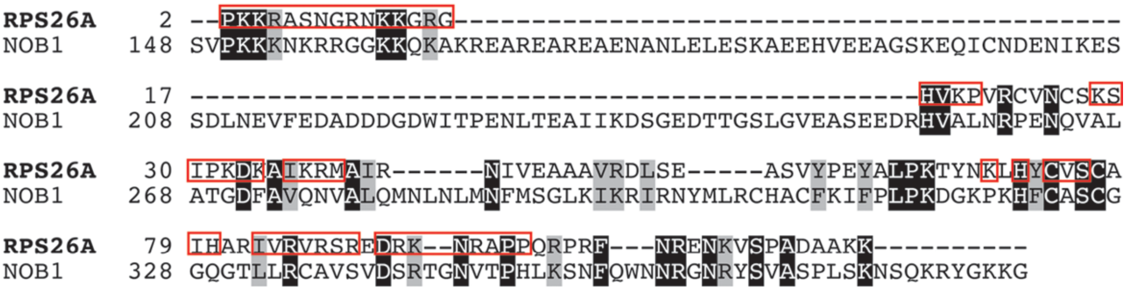

SUPPLEMENTARY FIGURE S2: Alignment of distinct ribosomal proteins and their respective ribosomal-like protein counterpart from *S. cerevisiae*. Sequence alignments were performed using the slow and accurate version of MAFFT [1]. Alignments were visualised with Belvu [2]. Identical residues are marked by black boxes and similar amino acids are marked by grey boxes (BLOSUM62). Red boxes indicate residues of the ribosomal proteins situated at or closer than 5 Å from rRNA. Comparison of the amino acid sequences of yeast (**A**) P0 (uL10), Mrt4 and Mex67, (**B**) L24 (eL24) and Rlp24, (**C**) L7 (uL30) and Rlp7, (**D**) S9 (uS4) and Imp3, (**E**) S26 (eS26) and Nob1. All sequences were taken from the *Saccharomyces Genome Database* (http://www.yeastgenome.org/).

Supplementary references

1. Kazutaka, K, Standley, DM (2013). Multiple Sequence Alignment Software Version 7: improvements in performance and usability. **Mol Biol Evol** 30(4): 772-780. doi: 10.1093/molbev/mst010.

2. Sonnhammer, ELL, Hollich, V (2005). *Scoredist*: A simple and robust protein sequence distance estimator. **BMC Bioinformatics** 6: 108. doi: 10.1186/1471-2105-6-108.
